# Supplementary material for: Protocol for the Tallaght University Hospital Institute for Memory and Cognition-Biobank for Research in Ageing and Neurodegeneration
Source: BMJ Open. 2023 Dec 8;13(12):e077772. doi: 10.1136/bmjopen-2023-077772 (PMC10729202; doi:10.1136/bmjopen-2023-077772)
Supplement: Supplementary data [file bmjopen-2023-077772supp001.pdf]

TIMC-BRAiN

TUH Institute for Memory & Cognition – Biobank for Research in Ageing & Neurodegeneration

Participant Information Leaflet (PIL)

Name of Biobank	TIMC-BRAiN Biobank
Location	Tallaght Institute of Memory and Cognition (TIMC), TUH
Principal Investigator (PI)	Professor Sean Kennelly, TUH
Co-Investigators	Dr Adam Dyer, TUH; Dr Helena Dolphin, TUH
Data Controller	Department of Age-Related Healthcare, TUH
Contact	Sean.Kennelly@tuh.ie; Adam.Dyer@tuh.ie; Helena.Dolphin@tuh.ie
Data Protection	Sean.Kennelly@tuh.ie; Adam.Dyer@tuh.ie; Helena.Dolphin@tuh.ie

You are invited to take part in the **TIMC-BRAiN Biobank**. This research study collects **clinical data** (including memory assessments, medical conditions and medications) and **biological samples** (including blood and cerebrospinal fluid samples), donated by participants for health research. This study helps doctors and researchers to learn more about the diagnosis and treatment of memory problems (such as cognitive impairment and dementia). This helps us to better understand how and why people develop these conditions. **Please read this leaflet carefully. Your physician in the Institute of Memory and Cognition in TUH can answer any questions that you may have.**

Key Words

When we say…	We mean…
Academic research	Research carried out in hospitals, universities, colleges and research institutes
Biobank	A collection of clinical data and biological samples donated by participants for health research
DNA	Your DNA (genes) make who you are by instructing cells to carry out specific tasks
Genetic Research	Research which examines peoples' genetic information (genes) to help understand how cognitive impairment and dementia develops and how best to treat it
Clinical Data	Information in your hospital chart, electronic patient record and other hospital databases including name, address, test results, images and scans.
Identifiable data	Information that might identify you such as name, address, date of birth
Non-identifiable ("Coded") data	Information that might identify you has been removed and replaced by a unique code. This is used instead of name, address, date of birth
Principal Investigator	The person responsible for a biobank or specific research study
Research Ethics Committee	Independent group of people who review each study to ensure that research is carried out ethically and safely and that rights are protected
Research studies	Research to learn more about cognitive impairment and dementia such as genetic research, how the condition changes or progresses, early detection and new diagnostics and treatments

PART 1 – THE STUDY

Why is this study being done?

Our research aims to learn more about **why people develop memory problems (such as cognitive impairment and dementia)**. We aim to understand **how and why** certain people develop these conditions. This includes analysis of **clinical information**, blood samples as well as lifestyle factors and family history. Additionally, we are trying to **develop new tests and methods of diagnosing these conditions**. This type of research requires many people to donate samples and data. This study (TIMC-BRAiN) speeds up research by allowing the research team have samples and data ready to use when needed. **The aim of this is to improve the diagnosis, management and treatment** of **memory problems (cognitive impairment** and **dementia).**

Your clinical data and biological samples will be stored **until researchers need them** in order to answer important questions. Your data and samples may thus be included in many different research studies and for this reason your data and samples may be stored indefinitely (forever). This biobank may share your **non-identifiable data** with other researchers working around the world. However, no potentially identifiable data will be shared outside of Tallaght University Hospital.

What happens if I take part ?

If you would like to take part, please read this leaflet and sign the consent form at the end. You will be given a copy of this leaflet to keep. A member of the research team will discuss this with you in more detail. If you are happy to take part, the biobank will collect and store

- **Clinical information** from your medical records, including medication history and medication usage in addition to test results from your cognitive assessment (memory tests)
- **Blood samples:** if you are having a routine blood test, the research doctor may take extra samples of blood to give to the biobank This is a small sample – about 3 tablespoons of blood
- **Cerebrospinal fluid samples**: if you are having a lumbar puncture as part of your memory assessment and diagnosis, the research team may ask to take an extra sample to store in the biobank. **This will only take place if you are having a lumbar puncture performed as part of your clinical assessment. There are no lumbar punctures being carried out solely for research purposes.**
- **Genetic (DNA) Samples**: DNA samples will be extracted from blood and samples for research. DNA is the molecule that provides instructions for how cells in our body work. By looking at DNA from people with and without vasculitis we may see differences that are important for diagnosis, treatment or understanding the condition.

What will happen to my clinical data and biological samples ?

All samples and data will be given a random study number in TUH by your study physician in a process called pseudo-anonymisation or "coding". This is intended to mask your identity. All biological samples will be stored in the Meath Foundation laboratory in Tallaght University Hospital using this code. Additionally, your clinical data will be stored on secure databases using this code. Only the study investigators have access to this code and this code will not be shared outside of the study team.

Samples will be stored in a freezer and will remain frozen for decades and potentially indefinitely (forever) so that we can identify future diagnostics and treatment for cognitive impairment and dementia. Samples are being kept indefinitely as scientific research rapidly changes and advances all the time, and we do not yet know what kinds of research questions will arise in the future. The only individuals with direct access to your samples are the investigators of the TIMC-BRAiN study.

Data and samples may be shared with other collaborators. If this is the case, a "Materials Transfer Agreement" will be drawn up by the TIMC-BRAiN study co-ordinators and the collaborating institutions/research groups. This may include samples and data being transferred outside of the European Economic Area (EEA). These samples and data will have all identifying features removed.

What are the benefits to me ?

Research can lead to better diagnostic tests, treatments and quality of life for people with cognitive impairment and dementia. The treatments that we have now developed are as a result of past research studies and biobanks. The TIMC biobank collects samples and healthcare data for future research and therefore we cannot predict at this time what research questions will arise in the future and what discoveries these will lead to. It takes many years to do research, so this research may not directly benefit you personally. **However, by participating you are contributing to improving the diagnosis, treatment and management of individuals living with cognitive impairment and dementia long into the future.**

What are the risks of participation ? Will I be informed of my results ?

As with any medical records, there is a theoretical risk of accidental disclosure of healthcare data. However, this is very unlikely as every effort will be made to protect your privacy (see below Section B and Section C). A blood sample may cause bruising or slight discomfort. The additional sample taken at time of diagnostic lumbar puncture poses minimal extra risk (you may be very slightly more likely to experience a post lumbar-puncture headache, however this additional risk is very minimal).

There are additional risks involved in genetic testing. Our genetic testing is entirely focused on the study of the genetics of cognitive impairment and dementia. Therefore it is possible

that even if you take part in the study. Even if we do not discover a genetic cause for your condition now, one may still be discovered in the future. It is common that we discover no genetic association for cognitive impairment or dementia or that it takes many years in order to do so. No results will be communicated as a result of genetic testing in this study as this is entirely a research study aimed at discovering genes for cognitive impairment and dementia. Thus, we are not testing for any other genetic mutations of potential clinical significance.

Do I have to take part ?

No, you do not have to take part. Your decision is completely voluntary and will not affect your medical care now or in the future.

What happens if I change my mind ?

You can change your mind at any time. If you contact the study team at any time to inform us that you no longer wish to participate, your samples will be **removed from the biobank and destroyed**. Additionally, the clinical data contributed as part of the biobanked will be removed from the biobank database. From that point on, none of your further samples of information will be used as part of the biobank. However, it may not be possible to destroy samples and healthcare data that have already been used for research as this could influence the results of the research.

What happens the results from research ?

Research is usually published in scientific and medical journals or presented at conferences so that other doctors and researchers can learn. You will not be identified in any of these publications or presentations. The research from TIMC-BRAiN will go on to inform the diagnosis, investigation and treatment of cognitive impairment and dementia into the future.

PART 2 – BIOBANK MANAGEMENT

How does the TIMC-BRAiN Biobank protect my privacy ?

TIMC-BRAiN has very strict governance procedures in place**. Only the TIMC-BRAiN team** have access to clinical data and biological samples. Only research studies that have been approved by the SJH/TUH JREC Ethics Committee will use clinical data and biological samples. Prof Kennelly and the study team must sign **legal agreements before clinical data and biological samples can be shared.** This ensures that your samples are only used as agreed. All members of the research team will undergo **General Data Protection Regulation (GDPR)** training and are bound by hospital confidentiality rules. TIMC-BRAiN ensures that your data is stored in a de-identified “coded” manner on **secure servers and only accessible by members of the study team**.

Has the biobank been approved by an ethics committee ?

Yes. This biobank has been approved by the SJH/TUH Joint Research Ethics Committee.

How is this biobank funded ?

This biobank is funded by departmental funds from the **Tallaght Institute of Memory and Cognition, Department of Age-Related Healthcare, Tallaght University Hospital**. The biobank does not make a profit from collecting or sharing your samples and healthcare data for health research.

Will I be paid for my involvement ?

No. You will not be paid to take part. It is hoped that your donation of samples and data will help other individuals undergoing investigation and treatment for cognitive impairment and dementia in the future.

PART 3 – WHAT DOES THE BIOBANK DO WITH MY HEALTHCARE DATA ?

Why does TIMC-BRAiN need to collect my clinical data ?

Clinical data is needed in order for researchers to choose the right biological samples for research or help interpret the results of their studies. To take part, you will be asked to share your clinical data collected as part of memory assessment and clinical workup with the TIMC-BRAiN biobank.

What type of clinical data will be collected ?

The biobank will collect healthcare and clinical data from your hospital chart, electronic records and other hospital databases. This data will only be accessed by study investigators (listed above). The study investigators (Dr Dyer/Dolphin, Prof Kennelly) may know your identity to follow your care and treatment. However, your identifiable information will only be able to be accessed by study investigators. The TIMC-BRAiN biobank will store the following clinical data:

- Name, gender, date of birth, hospital number, laboratory identifiers; this information will be stored on a protected database **only accessible by the study investigators**
- Results of blood tests, medical history, regular medications, lifestyle information (smoking, alcohol use), neuroimaging results and final diagnosis and treatment. This information is all routinely obtained as part of work-up, assessment, diagnosis and treatment of cognitive impairment and dementia.
- Results of cerebrospinal fluid investigations, if applicable.

How is my data stored and shared ?

When you are enrolled in the study, you will be assigned an anonymous study number. The "key" will be stored in TUH on a locked database only accessible to the study investigators. Clinical data and clinical results will be stored on a secure database maintained on servers in Tallaght University Hospital. This will be stored using the REDCap system (Research Electronic Data Capture) which is used globally for the storage of sensitive research data. This will only be accessible by study investigators.

Your clinical data may be shared as:

(i) **Identifiable Data**: the study investigators at the Tallaght Institute of Memory and Cognition may know your identity in order to follow your care and treatment. Only the above named investigators will have access to your identifiable data

(ii) **Non-identifiable data:** researchers working as part of collaborations or research studies outside of the study team (either in Trinity College Dublin, academic partners of TUH, or as part of other researchers) receive "non-identifiable data". This means that information that may identify you has been removed and replaced with a unique code which is used instead of your hospital number, date of birth, address. These researchers work in universities or hospital. Data sharing will only take place as outlined above and your data will not be shared with any external companies or for-profit organisations.

What are my rights ?

TIMC-BRAiN has data protection measures in place and is committed to ensuring that your rights under GDPR (General Data Protection Regulation) are protected. The Department of Age-Related Healthcare is the data controller for this study. We also ask for your consent as a data protection safeguard, in accordance with the Irish Health Research Research Regulations 2018.

- Under the European General Data Protection Regulation (GDPR), the lawful basis for processing your data in this study is for scientific research (Article 9(2) (j)) in the public interest (Article 6(1)(e)).
- Under GDPR, you can exercise the following rights in relation to your personal data, unless the request would make it impossible or very difficult to conduct the research:
  - The right to access to your data and receive a copy of it
  - The right to restrict or object to processing of your data
  - The right to object to any further processing of the information we hold about you (except where it is de-identified)
  - The right to have inaccurate information about you corrected or deleted

    - The right to receive your data in a portable format and to have it transferred to another data controller
    - The right to request deletion of your data
- You can exercise these rights be contacting your study doctor/study investigator
- Under GDPR, if you are not satisfied with how your data is being used, you have the right to lodge a complaint with the Data Protection Commissioner of Ireland or the study Data Protection Officer (dpo@tuh.ie)

TIMC-BRAiN

TUH Institute for Memory & Cognition – Biobank for Research in Ageing & Neurodegeneration

CONSENT FORM

To be completed by the participant:

I have read and understood the information leaflet for the study. The information has been fully explained to me and I have been able to ask questions, all of which have been answered to my satisfaction	YES ☐	NO ☐
I have had the opportunity to discuss the study, ask questions about the study and I have received satisfactory answers to all my questions.	YES ☐	NO ☐
I understand that my participation is voluntary, and I can withdraw my biological material and data at any time without giving a reason. I understand that opting out will not affect my future medical care.	YES ☐	NO ☐
I understand that sections of my medical notes may be looked at by study investigators at Tallaght Hospital. I give permission for these individuals to access my records. All information will be kept private and confidential.	YES ☐	NO ☐
I agree for the entry of my clinical data into the registry. I give explicit informed consent for my data to be processed as part of this research study. I understand that my data will be securely coded and stored indefinitely.	YES ☐	NO ☐
I agree to provide blood and (if applicable) cerebrospinal fluid samples for this study as described in the information leaflet. I understand that my samples will be securely coded and stored indefinitely. The risk of taking samples has been explained.	YES ☐	NO ☐
I consent for my biological coded samples (including DNA) to be shared with authorised third parties including national and international institutions and academic institutions. I understand that this requires a legal materials transfer agreement between researchers at TUH and these institutions.	YES ☐	NO ☐
I agree to be contacted by researchers as part of this study	YES ☐	NO ☐
I consent to undergo venepuncture for the purposes of this study	YES ☐	NO ☐
I consent to participation in this research study having been fully informed of the risks, benefits and purpose of the study	YES ☐	NO ☐
I consent to be contacted with regard to the possible use of my data and/or biological material for future research studies (with consent)	YES ☐	NO ☐
I understand that processing of my personal data, including transfer of data outside the EU, will be protected in accordance with the General Data Protection Regulation	YES ☐	NO ☐
I understand that results from the analysis of my samples will not be given to me. I understand that there are no direct benefits to me and I will not benefit financially in any way.	YES ☐	NO ☐

Participant's Name (Block Capitals):	
Participant's Signature:	
Date:	

To be completed by the **RESEARCHER**:

I have fully explained the purpose and nature (including benefits and risks) of this study to the participant in a way that he/she could understand. I have invited him/her to ask questions on any aspect of the study.	YES ☐	NO ☐
I confirm that I have given a copy of the information leaflet and consent form to the participant and family member/carer.	YES ☐	NO ☐

Researcher's Name (Block Capitals):	
Researcher's Title & Qualifications:	
Researcher's Signature:	
Date:	
