# Supplementary material for: Protocol for the Tallaght University Hospital Institute for Memory and Cognition-Biobank for Research in Ageing and Neurodegeneration
Source: BMJ Open. 2023 Dec 8;13(12):e077772. doi: 10.1136/bmjopen-2023-077772 (PMC10729202; doi:10.1136/bmjopen-2023-077772)
Supplement: Author's manuscript [file bmjopen-2023-077772.draft_revisions.pdf]

BMJ Open

BMJ Open is committed to open peer review. As part of this commitment we make the peer review history of every article we publish publicly available.

When an article is published we post the peer reviewers' comments and the authors' responses online. We also post the versions of the paper that were used during peer review. These are the versions that the peer review comments apply to.

The versions of the paper that follow are the versions that were submitted during the peer review process. They are not the versions of record or the final published versions. They should not be cited or distributed as the published version of this manuscript.

BMJ Open is an open access journal and the full, final, typeset and author-corrected version of record of the manuscript is available on our site with no access controls, subscription charges or pay-per-view fees (http://bmjopen.bmj.com).

If you have any questions on BMJ Open's open peer review process please email info.bmjopen@bmj.com

BMJ Open

Tallaght University Hospital Institute for Memory and Cognition – Biobank for Research in Ageing and Neurodegeneration (TIMC-BRAiN): Study Protocol

Journal:	*BMJ Open*
Manuscript ID	bmjopen-2023-077772
Article Type:	Protocol
Date Submitted by the Author:	14-Jul-2023
Complete List of Authors:	Dyer, Adam; Tallaght University Hospital, Institute of Memory and Cognition; Trinity College Dublin, Department of Medical Gerontology, School of Medicine<br>Dolphin, Helena; Tallaght University Hospital, Institute of Memory and Cognition; Trinity College Dublin, Department of Medical Gerontology, School of Medicine<br>O'Connor, Antoinette; Tallaght University Hospital, Department of Neurology<br>Morrison, Laura; Tallaght University Hospital, Institute of Memory and Cognition; Trinity College Dublin, Department of Medical Gerontology, School of Medicine<br>Sedgwick, Gavin; Tallaght University Hospital, Institute of Memory and Cognition<br>McFeely, Aoife; Tallaght University Hospital, Institute of Memory and Cognition; Trinity College Dublin, Department of Medical Gerontology, School of Medicine<br>Killeen, Emily; Tallaght University Hospital, Institute of Memory and Cognition<br>Gallagher, Conal; Tallaght University Hospital, Institute of Memory and Cognition<br>Davey, Naomi; Tallaght University Hospital, Institute of Memory and Cognition<br>Connolly, Eimear; Tallaght University Hospital, Institute of Memory and Cognition<br>Lyons, Shane; Tallaght University Hospital, Department of Neurology<br>Young, Conor; Tallaght University Hospital, Institute of Memory and Cognition<br>Gaffney, Christine; Tallaght University Hospital, Department of Neurology<br>Ennis, Ruth; Tallaght University Hospital, Institute of Memory and Cognition<br>McHale, Cathy; Tallaght University Hospital, Institute of Memory and Cognition<br>Joseph, Jasmine; Tallaght University Hospital, Institute of Memory and Cognition<br>Knight, Graham; Tallaght University Hospital, Institute of Memory and Cognition<br>Kelly, Emmet; Tallaght University Hospital, Institute of Memory and Cognition

O'Farrelly, Cliona; Trinity College Dublin, Comparative Immunology
Bourke, Nollaig M; Trinity College Dublin, Department of Medical Gerontology, School of Medicine
Fallon, Aoife; Tallaght University Hospital, Institute of Memory and Cognition; Trinity College Dublin, Department of Medical Gerontology, School of Medicine
O'Dowd, Sean; Tallaght University Hospital, Department of Neurology; Trinity College Dublin, Academic Unit of Neurology
Kennelly, Sean P; Trinity College Dublin, Department of Medical Gerontology, School of Medicine; Tallaght University Hospital, Institute of Memory and Cognition

Keywords: Dementia < NEUROLOGY, Delirium & cognitive disorders < PSYCHIATRY, GERIATRIC MEDICINE

SCHOLARONE™
Manuscripts

Tallaght University Hospital Institute for Memory and Cognition – Biobank for Research in Ageing and Neurodegeneration (TIMC-BRAiN): Study Protocol

Adam H Dyer[1-2†], Helena Dolphin[1-2*†], Antoinette O'Connor[3], Laura Morrison[1-2], Gavin Sedwick[1], Aoife McFeely[1-2], Emily Killeen[1], Conal Gallagher[1], Naomi Davey[1], Eimear Connolly[1], Conor Young[1], Shane Lyons[3], Christine Gaffney[3], Ruth Ennis[1], Cathy McHale[1], Jasmine Joseph[1], Graham Knight[1], Emmet Kelly[3], Cliona O'Farrelly[5], Nollaig M Bourke[2], Aoife Fallon[1,2], Sean O'Dowd[3,4], & Sean P Kennelly[1,2]

1. Institute of Memory and Cognition, Tallaght University Hospital, Dublin, Ireland
2. Department of Medical Gerontology, School of Medicine, Trinity College Dublin, Ireland
3. Department of Neurology, Tallaght University Hospital, Dublin, Ireland
4. Academic Unit of Neurology, Trinity College Dublin, Ireland
5. Comparative Immunology, Trinity Biomedical Sciences Institute, Trinity College Dublin, Ireland

[†]Authors Contributed Equally to This Work

[*]Corresponding Author

Dr Adam Dyer

Department of Age-Related Healthcare

Tallaght University Hospital

Dublin, Ireland

E-mail: dyera@tcd.ie

Telephone: 00353-1-4142000

Running Title: TIMC-BRAIN Protocol

Key Words: Alzheimer Disease, Dementia, Cerebrospinal Fluid, Blood-Based Biomarkers, Neuroimaging

Abstract Word Count: 299

Main Text Word Count: 3,898

For peer review only

Abstract

Introduction: Alzheimer's Disease (AD) and other dementias affect >50 million individuals globally and are characterised by broad clinical and biological heterogeneity. Cohort and biobank studies have played a critical role in advancing understanding of disease pathophysiology and in identifying novel diagnostic and treatment approaches. However, further discovery and validation cohorts are required to clarify the real-word utility of new biomarkers, facilitate research into the development of novel therapies and to advance our understanding the clinical heterogeneity and pathobiology of neurodegenerative diseases.

Methods and Analysis: The Tallaght University Hospital Institute for Memory and Cognition Biobank for Research in Ageing and Neurodegeneration (TIMC-BRAiN) will recruit 1,000 individuals over 5 years. Participants, who are undergoing diagnostic work-up in the TIMC Memory Assessment and Support Service (TIMC-MASS), will opt to donate clinical data and biological samples into a biobank. All participants will complete a detailed clinical, neuropsychological and dementia severity assessment (including ACE: Addenbrooke's Cognitive Assessment/RBANS: Repeatable Battery for Assessment of Neuropsychological Status/CDR: Clinical Dementia Rating Scale). Participants undergoing venepuncture/lumbar puncture as part of clinical work-up will be offered the opportunity to donate additional blood (serum/plasma/whole blood) and cerebrospinal fluid (CSF) samples for longitudinal storage in the TIMC-BRAIN biobank. Participants are followed at 18-month intervals for repeat clinical and cognitive assessment. Anonymised clinical data and biological samples will be stored securely in a central repository and used to facilitate future studies concerned with advancing with the diagnosis and treatment of neurodegenerative diseases.

Ethics and Dissemination: Ethical Approval has been granted by the St James's Hospital (SJH)/Tallaght University Hospital (TUH) Joint Research Ethics Committee (JREC) [Project ID: 2159] which operates in compliance with the European Communities (Clinical Trials on Medicinal Products for Human Use) Regulations 2004 and ICH Good Clinical Practice Guidelines. Findings using TIMC-BRAiN will be published in a timely and open-access fashion.

For peer review only

Strengths and Limitations of This Study

- TIMC-BRAiN, the first cognitive biobank in Ireland, will prospectively recruit 1,000 individuals undergoing assessment for cognitive symptoms obtaining clinical data and biological samples that will be stored in a comprehensive, secure biobank. This will facilitate future studies aimed at elucidating the underlying disease biology, diagnostic and treatment approaches in cognitive impairment and dementia.
- Comprehensive clinical assessment is based on gold-standard cognitive and neuropsychological assessment, appropriate clinical work-up with final biobank diagnosis adjudicated by multi-disciplinary consensus in accordance with published guidelines with input from geriatric medicine, neurology, radiology, nursing, speech and language therapy, occupational therapy and neuropsychology colleagues.
- Biological samples donated by TIMC-BRAiN participants include serum, plasma, cerebrospinal fluid (including cell-free cerebrospinal fluid and immune cell pellet where diagnostic lumbar puncture performed), whole blood for later genetic analysis and whole blood stored in Cytodelics™ stabiliser. All samples are anonymised stored in a central repository coupled to comprehensive clinical data and are available for future research studies.
- Participants are assessed every 18-months for clinical progression, including progression to established cognitive impairment/dementia in those with early cognitive symptoms and disease severity progression in those with established dementia.
- TIMC-BRAiN is a single-site study, however the Memory Assessment and Support Service (MASS) at Tallaght University Hospital (TUH) accepts national referrals and is the largest MASS in Ireland.

For peer review only

Introduction

The global prevalence of dementia is expected to sharply increase over the coming decades, affecting >150 million individuals globally by 2050 (1, 2). Recent decades have seen significant advances in our understanding of the neurobiology of dementia, in particular dementia due to Alzheimer's Disease (AD). However, there remains an urgent need to advance understanding of the pathobiology of all dementia syndromes. Such progress will be critical to the discovery of novel diagnostic and prognostic markers, as well as advancing understanding of their real-world utility. In addition, further understanding of clinical and biological phenotypes and underlying pathophysiological processes is an urgent priority for the field to enable better approaches to personalised prevention and treatment, particularly once Disease Modifying Treatments (DMTs) become available.

Neurodegenerative diseases have high degrees of phenotypic, genetic and pathophysiological heterogeneity with traditional diagnostic paradigms centred on late-stage syndromic classification of disease phenotypes (3). Importantly, personalised approaches to clinical-biological classification incorporating comprehensive assessment of clinical phenotype accompanied by the use of appropriate neuro-imaging, biological sampling (such as cerebrospinal fluid (CSF) biomarkers) and further diagnostic tests serves to map observed clinical phenotypes onto known neurobiological substrates (4, 5). An accurate (and timely) diagnosis has important implications for treatment, advance care planning and is valued by those living with neurodegenerative conditions (6, 7).

Many Memory Assessment and Support Services (MASS) use diagnostic biomarkers to assist in the diagnosis and in prognostication of individuals with cognitive impairment. In AD, CSF markers of amyloid and tau pathology are now frequently employed in many MASS (8). Lumbar Puncture [LP] is typically well-tolerated in these settings (9). Additionally, the recent availability of amyloid and tau Positron Emission Tomography (PET) in certain jurisdictions allows non-invasive assessment of AD pathology (10, 11). Progress in the field of biomarker development has not been solely confined to the field of AD. Promising biomarkers that may aid in the diagnosis of other neurodegenerative disorders are also being developed – for instance the identification of isoform-specific tau species in primary

For peer review only

tauopathies such as Cortico-Basal Degeneration (CBD) and Progressive Supranuclear Palsy (PSP) and the detection of alpha-synuclein in CSF by RT-QuiC in prodromal Parkinson's disease and Dementia with Lewy Bodies (DLB) (12, 13).

One of the most exciting advances in the field of neurodegenerative disorders has been the advent of blood-based biomarkers. At present, blood-based biomarkers– exemplified by research in AD - show remarkable promise, even in pre-symptomatic disease stages (14-19). These advances will be accompanied by significant challenges in the implementation and interpretation of these biomarkers in clinical practice. Importantly, validation of these biomarkers in clinical populations, with consideration of issues such as renal clearance, medical comorbidity and population-specific norms, will require the use of large validation cohorts prior to widespread implementation.

A key priority in dementia research is understanding clinical and biological phenotypes across the disease spectrum, which can aid in accurate diagnosis, prognostication and treatment plans for those affected. For instance, Mild Cognitive Impairment (MCI) is characterised by deficits evident on neuropsychological testing which do not interfere with day-to-day function and may stabilise, progress to dementia or even revert to normal cognition over time (20). Around 5-15% of individuals with MCI progress to dementia annually (21). One of the greatest challenges at present is predicting the variable disease trajectory in those affected by MCI and there is an urgent need for new clinical, diagnostic and biological markers that may indicate a greater likelihood of disease progression. Whilst several notable studies have demonstrated the influence of AD biomarkers on disease progression in MCI, further studies are needed to establish the optimal use of prognostic markers in individuals with early cognitive impairment (22)

The development of large-scale clinical, imaging, genetic and biological repositories and real-world clinical cohorts are crucial to the discovery, identification, validation and standardisation of clinical and biomarker-based assessments for AD and other neurodegenerative conditions (15, 23-26). Longitudinal observational cohort studies are integral to advancing understanding of relationship between clinical and fluid biomarkers, cognition and clinical progression across all neurodegenerative diseases, especially as we enter an era of disease modifying therapies (27). The development of

For peer review only

further large cohorts is essential in facilitating the translation of biomarker-based discoveries into clinical practice and further research into the underlying pathobiology and treatment of neurodegenerative conditions.

The Tallaght University Hospital Institute of Memory and Cognition Biobank for Research in Ageing and Neurodegeneration (TIMC-BRAiN) will create a longitudinal biobank of clinical data and biological samples in individuals undergoing assessment for memory and cognitive symptoms at TIMC-MASS. This biobank will facilitate research into clinical and biological biomarkers, in addition to research studies focused on the underlying neurobiology of MCI, dementia and other neurodegenerative conditions.

For peer review only

Aims

TIMC-BRAiN is a longitudinal biobanking study commencing in January 2023 that will recruit 1,000 individuals attending the TIMC-MASS for workup and assessment of cognitive symptoms. TIMC-BRAiN will biobank longitudinal clinical and neuroimaging data alongside biological samples. This biobank will serve as a repository for future research studies that seek to (i) advance understanding of disease pathobiology, (ii) evaluate the use of diagnostic and prognostic tests/assessments and (iii) improve precision medicine approaches across neurodegenerative diseases.

The aims of TIMC-BRAIN are as follows:

- To create a clinical data, neuroimaging and biological sample repository from individuals being assessed for concerns relating to cognition at the TIMC MASS.
- To record final diagnosis and comprehensive clinical phenotyping in those recruited - including demographic information, medical history, cardiovascular risk factors, family history, specific cognitive symptoms (for instance episodic memory/autobiographical memory, language, facial recognition, topographical memory), neuropsychological symptoms, mobility/gait, sleep, nutrition, mood, frailty, hearing and vision.
- To record neuroimaging results coupled to clinical data consisting of Magnetic Resonance Imaging (MRI) or Computed Tomography (CT) Brain results with scoring of MRI scans for vascular burden, parietal atrophy and medial temporal lobe atrophy in addition to results of nuclear imaging and other scans performed where clinically appropriate.
- To biobank biological samples including peripheral blood (whole blood for DNA, serum, plasma and whole blood stored in blood stabiliser) and CSF– including both CSF supernatant and cryopreserved immune cells.
- To longitudinally track changes in cognition (via repeat cognitive assessment) and conversion to/progression of dementia at subsequent 18-month follow-up visits, performed alongside routine clinical care.

For peer review only

As TIMC-BRAiN is a longitudinal clinical data and biological sample repository, there are no pre-specified hypotheses to be tested. TIMC-BRAiN will afford a ready clinical and biological repository for important research questions to be answered in the future and aims to collaborate widely to enable novel basic biological and translational research aimed at improving diagnosis, prognostication and treatments for those affected by neurodegenerative diseases.

For peer review only

Methods

Study Setting & Design

Tallaght University Hospital (TUH) is a tertiary referral hospital in Dublin, Ireland with a catchment area of nearly 500,000 individuals. The TUH Institute of Memory and Cognition (TIMC) is the largest MASS in Ireland and receives referrals from both TUH catchment and nationally for individuals experiencing cognitive symptoms. Approximately 400-500 patients are assessed annually in the TIMC-MASS. Once referred to the service, patients are assessed in the first instance by an Advanced Nurse Practitioner (ANP). Comprehensive assessment includes detailed neuropsychological assessment, medical assessment, routine blood tests and appropriate neuroimaging before each case is individually discussed at a weekly Multi-Disciplinary Team (MDT) consensus meeting where working diagnosis is discussed and further investigations if needed, are advised.

Inclusion and Exclusion Criteria

TIMC-BRAiN will recruit participants undergoing assessment in the TIMC-MASS (See Figure 1). TIMC-BRAiN will recruit a diverse cohort including individuals with different levels of cognitive performance - Subjective Memory Concerns (SMCs), Mild Cognitive Impairment (MCI), and dementia - and a representative array of neurodegenerative diseases - to include AD, Lewy Body Disease (LBD), Frontotemporal Dementia (FTD) and other neurodegenerative conditions. In order to reflect a real-world clinical cohort, participants will not be excluded from TIMC-BRAiN based on age or medical comorbidity. However, individuals with severe systemic illness such as malignancy with limited life expectancy, individuals with current significant alcohol or substance misuse or those with current significant psychiatric comorbidity will be excluded from participation.

<Insert Figure 1>

Participants will be provided with a Participant Information Leaflet (PIL) at time of routine clinical assessment in the TIMC and allowed to reflect on whether they wish to donate their

For peer review only

clinical/neuroimaging data and biological samples in the TIMC-BRAiN biobank. Participants are informed that participation is entirely voluntary and will not in any way affect their future clinical care. Potential participants will then be approached after having time to reflect on participation at time of phlebotomy/lumbar puncture by a study investigator/sub-investigator to discuss participation. Figure 1 outlines assessment and recruitment pathway.

Clinical and neuropsychological Assessment

Clinical and cognitive assessments are performed by an Advance Nurse Practitioner (ANP) as part of routine care at the TIMC – the contents of the TIMC Case Report Form (CRF) are given in Table 1. Information collected includes background/demographic information, medical history, regular medications, hearing and vision, smoking status (yes/no/previous) and family history (detailed in Table 1).

Table 1. Information Recorded on TIMC-BRAiN Case Report Form [CRF]

Item	Data Recorded
**Demographic Information**	
Age	Age at Assessment; Years
Sex	Biological Sex; Male/Female/Non-Binary
Level of Education	Finished Formal Education; Years
Occupation	Occupation; Free Text
Age Retired	Age at Retirement; Years
**Medical History**	
Prior Stroke/Transient Ischaemic Attack	Yes/No
Recurrent Syncope	Yes/No
Diabetes Mellitus (5)	History of/on DM medication; Yes/No
Hypertension	History of/on antihypertensive medication; Yes/No
Hypercholesterolaemia	History of/on anti-lipidaemic medication; Yes/No
Ischaemic Heart Disease	Yes/No
Alcohol Excess	Yes/No
Epilepsy	History of/on anti-epileptic medication; Yes/No
Concussion/Prior Head Injury	Yes/No
History of Malignancy	Yes/No
Previous Anxiety	History of/on anxiolytic medication; Yes/No
Previous Depression	History of/on antidepressant medication; Yes/No
Hearing Impairment	Yes/No
Vision Impairment	Yes/No
Smoking Status	Yes/No/Previous Smoker
Family History	Memory Difficulties/Dementia/Alzheimer's Disease/ in a 1st degree relative; Yes/No
Regular Medications	Medications List; Coded Using Anatomic Therapeutic Classification (ATC) system
Anosmia	Yes/No
**Cognitive Symptoms at Presentation**	
Symptom Duration	Duration of Symptoms; in Months
Episodic Memory<br>• Lose Track of Days<br>• Forgetting Appointments<br>• Misplacing Objects<br>• Forgetting to Pay Bills<br>• Forgetting How to Use Appliances<br>• Remembering to Take Medications	Each Item Recorded as Yes/No Indicating Recent Change at Time of Assessment

For peer review only

Autobiographical Memory<br>• Forgetting Past Personal Events	Recorded as Yes/No Indicating Recent Change at Time of Assessment
Language<br>• Word-Finding Difficulties<br>• Shrinkage of Vocabulary<br>• Comprehending Speech<br>• Comprehending Written Information<br>• Ability to Engage in Conversations	Each Item Recorded as Yes/No Indicating Recent Change at Time of Assessment
Recognition<br>• Facial Recognition<br>• Getting Lost in Familiar Areas<br>• Changes in Using Public Transport/Driving	Each Item Recorded as Yes/No Indicating Recent Change at Time of Assessment
Informant Concerns<br>• Issues with Cooking<br>• Issues with Orientation<br>• Issues with Driving<br>• Issues with Medication Compliance<br>• Recent Falls	Each Item Recorded as Yes/No Indicating Recent Change at Time of Assessment
**Cognitive Assessment**	
RBANS:<br>• Index I (Immediate Memory)<br>• Index II (Visuospatial/Constructional)<br>• Index III (Language)<br>• Index IV (Attention)<br>• Index V (Delayed Memory)<br>• Global Score	Repeatable Battery for Assessment of Neuropsychological Status (RBANS)<br>Unadjusted (Raw) Score and Centile Score Computed (Based on Age/Education)<br>Normalisation using Duff Norms
ACE-III<br>• Attention<br>• Memory<br>• Fluency<br>• Language<br>• Visuospatial<br>• Overall Score	Addenbrooke's Cognitive Assessment (ACE-III) if Unable to Complete RBANS<br>Domain and Total Scores Recorded
CDR<br>• Memory<br>• Orientation<br>• Judgement & Problem Solving<br>• Community Affairs<br>• Home and Hobbies<br>• Personal Care	Clinical Dementia Rating Scale<br>Global Score and Sum of Boxes Recorded
FAB	Frontal Assessment Battery<br>Total Score Recorded
AD8 Administered to Informant	Ascertain-Dementia 8 Questionnaire (Scored from 8)
CBI-R	Cambridge Behavioural Inventory Revised (CBI-R)
**Mood & Anxiety Assessment**	
HADS-Depression<br>HADS-Anxiety	Hospital Anxiety and Depression Scales<br>Total Score Recorded<br>Probable Depression/Anxiety (Score >10) Recorded
**Further Clinical Assessment**	
PSQI	Pittsburgh Sleep Quality Index (0-21)
TUG	Timed-Up-And-Go Test; Time to Complete in seconds recorded
CFS	Clinical Frailty Scale (CFS) to Assess Frailty
MNA	Mini Nutritional Assessment Score (0-7: Malnourished; 8-11: At Risk; 12-14: Normal)
**Neuroimaging**	
MRI Brain<br>• Fazekas Score<br>• MTA Score<br>• Koedam Score	Adjudicated at MDT Consensus Meeting by a panel of >2 geriatricians/neurologists<br>• Fazekas Score 0-3 for White Matter Disease<br>• Medial Temporal Atrophy (MTA) score 0-4<br>• Koedam Score for Parietal Atrophy score 0-3
CT Brain Results	CT Brain results if performed; free text
FDG-PET Results	FDG-PET results if performed; free text
DAT Results	DAT scan results if performed; free text
**Blood Test Results**	
Haematology Results<br>• Full Blood Count (FBC)	Any abnormalities detected; yes/no
Biochemistry & Other Results<br>• Renal Profile<br>• Liver Profile<br>• Bone Profile<br>• HbA1c	Any abnormalities detected; yes/no

For peer review only

• Lipid Profile<br>• Micronutrients: Vitamin B12, Folate<br>• Vitamin D	
**Cerebrospinal Fluid Results**	
Diagnostic Lumbar Puncture Results<br>• $A\beta_{1\text{-}42}$<br>• T-Tau<br>• P-Tau	All recorded as pg/mL using Roche Elecsys Electrochemiluminesence Assay
**Working (Consensus Meeting) Diagnosis**	
Working Diagnosis at Weekly MDT Meeting	Free Text
**Final (Biobank) Diagnosis**	
Final Diagnosis Adjudicated Following all Investigations	Functional Status:<br>• Subjective Memory Complaints<br>• Mild Cognitive Impairment<br>• Dementia<br>Aetiological Diagnosis:<br>• Alzheimer's Disease (amnestic, behavioural, LPA, CBS, and PCA variants)<br>• Lewy Body Disease<br>• Frontotemporal Dementia (FTD) (bvFTD/ nfvPPA/ svPPA)<br>• FTD overlap syndromes: FTD-ALS/FTD-CBS/FTD-PSP<br>• Corticobasal Syndrome: 4R Tauopathy<br>• Progressive Supranuclear Palsy<br>• Vascular Cognitive Impairment/Dementia<br>• Other Diagnosis [Free Text]<br>• Genetic Diagnosis [Free Text]

**bvFTD: behavioural variant FTD; LPA: Logopenic variant aphasia, CBS: corticobasal syndrome, PCA: Posterior cortical atrophy: nfvPPA: non fluent agrammatic PPA, svFTD: semantic variant FTD; 4R Tau: 4 repeat tauopathy: PPA: primary progressive aphasia*

All participants undergo comprehensive clinical history, a multi-domain cognitive assessment and a collateral history is obtained for all participants which specifically examines driving safety, medication compliance in addition to classifying duration and domains of cognitive change. Cognitive symptoms are assessed by recent changes in the following domains: (i) episodic memory, (ii) autobiographical memory (iii) language and (iv) facial recognition/topographical memory. All of these variables are recorded as yes/no to indicate a recent change. Total duration of symptoms (in months) and first predominant symptom is also recorded.

Neuropsychological assessment consists of the Addenbrooke's Cognitive Examination (ACE-III) (28) and Frontal Assessment Battery (FAB) (29) – these items are completed in all participants Additionally the Repeatable Battery for the Assessment of Neuropsychological Status (RBANS) is performed if possible (30). The Clinical Dementia Rating Scale (CDR) global/sum of boxes is used to assess for dementia severity (31). The Ascertain Dementia 8 (AD8) questionnaire is also routinely administered to informants (32). The Cambridge Behavioural Inventory (CBI) captures recent behavioural changes and cognitive changes and affective symptoms are reported (33).

For peer review only

Mood and Anxiety are routinely assessed via the Hospital Anxiety and Depression Scale (HADS) Anxiety and Depression scales (34). Sleep is assessed via the Pittsburgh Sleep Quality Index (PSQI) (35). Mobility is assessed by the Timed-Up-And-Go (TUG) test (36). Frailty is assessed via the Clinical Frailty Scale (CFS) (37) . Nutrition is assessed in all participants using the Mini-Nutritional Assessment (MNA) (38). The results of all these assessments are recorded in the TIMC-BRAIN Case Report Form.

Neuroimaging

All individuals assessed in the TIMC have an MRI Brain performed, unless contra-indicated. MRI scans are reviewed at weekly consensus meeting and the following scores applied:

(i) **Fazekas Score**: grades white matter disease, scored from 0-3 (39)

(ii) **Medial Temporal Lobe Atrophy (MTL):** grades mesio-temporal atrophy, scored from 0-4 (40)

(iii) **Parietal Atrophy Score (Koedam Score):** grades parietal atrophy, scored from 0-3 (41)

Scores are applied by a panel of at least two consultants in geriatric medicine and neurology. These scores are recorded in TIMC-BRAiN data repository. Some individuals proceed to having additional imaging e.g. fluorodeoxyglucose (FDG)-PET or Dopamine Uptake (DaTScan) scans. The outcome of all relevant imaging is documented within the data repository (See Table 1).

Diagnostic Workflow in TIMC-MASS & Final TIMC-BRAIN Diagnosis

All cases, after completion of their clinical, neuropsychological and neuroimaging assessments, are discussed at a weekly consensus diagnostic meeting. This meeting, led by a consultant geriatrician/neurologist with expertise in neurodegenerative diseases, determines, where possible, a working (consensus) diagnosis. Further investigations such as advanced neuroimaging or diagnostic CSF sampling are recommended in appropriate cases.

For peer review only

For individuals recruited into TIMC-BRAIN a separate biobank diagnostic meeting occurs on a bi-monthly basis. This meeting is convened by the biobank co-ordinator and attended by the biobank co-ordinator, data manager, research fellows, consultant geriatrician, consultant neurologist and study sub-investigators. Each case is discussed only after all appropriate clinical investigations/assessments have taken place and a final "biobank" diagnosis is confirmed reflecting (i) functional status and (ii) aetiological diagnosis as follows:

Functional status :

- Subjective Memory Complaints (SMCs): individuals with concerns over cognition/memory symptoms, free from established cognitive impairment on neuropsychological testing
- Mild Cognitive Impairment (MCI): impairments on 1 or more neuropsychological domain [typically 1.5 standard deviation fromage/education mean], which do not interfere with day-to-day function. (20)
- Dementia: objective cognitive loss of any domain which is severe enough to cause functional decline in day-to-day activities.

Aetiological Diagnosis:

Published consensus criteria are used to classify aetiology as:

- AD (subtyped into amnestic, behavioural, logopenic variant aphasia, corticobasal syndrome, and posterior cortical atrophy variants) (5, 42, 43)
- Lewy Body Disease (44)
- Frontotemporal Dementia (FTD) subtyped into behavioural variant FTD (bvFTD), semantic variant FTD (svFTD), non-fluent agrammatic FTD (nfvFTD) (45) (46). Also, where applicable, include overlap syndromes: FTD-Corticobasal Syndrome (CBS); FTD-PSP (Progressive Supranuclear Palsy); FTD-ALS (Amytrophic Lateral Sclerosis)
- Corticobasal Syndrome (CBS) – 4R tauopathy (47, 48)
- Progressive Supranuclear Palsy (PSP) (49)
- Vascular Cognitive impairment/Dementia (50)

For peer review only

- Other Diagnosis: This includes individuals with a diagnosis which does not conform to one of the above categories. Details around potential diagnoses will be recorded as free-text by the biobank manager.
- Genetic diagnosis. For the subset of participants with a genetic diagnosis this will also be reordered.

In cases where participants meet criteria for two aetiological diagnosis, e.g. nfvPPA and FTD-PSP, both aetiological diagnoses will be recorded as the final TIMC-BRAIN diagnosis. The biobank co-ordinator is responsible for recording the final biobank diagnosis on the appropriate participants RedCAP diagnostic section (see below under "Data Storage").

Blood and Cerebrospinal Fluid Sampling

Following informed consent, blood sampling will be performed as part of routine phlebotomy if possible but in certain cases, may need to be performed separately. All blood tests will be obtained between 08:00-12:00 to minimise the risk of diurnal variation on biomarkers and processed on-site at TIMC within 2 hours of blood draw. For TIMC-BRAIN participants, a serum clot activator tube (9mL), two EDTA tubes (9mL each) and a Lithium Heparin (3mL) coated tube are collected. Fasting status will be recorded – however, participants are not asked to fast for the purposes of TIMC-BRAiN participation.

Individuals undergoing diagnostic CSF examination as part of their clinical assessment will be offered the opportunity to donate CSF samples to the TIMC-BRAiN biobank by a study investigator/sub-investigator. LPs are performed between 09:00-12:00 and are carried out in standard aseptic fashion. LPs are performed at the L3-L5 level and manometers are avoided the maximum number of attempts (including change of operator where required) is 3. Diagnostic samples are obtained in the first instance, followed by an additional 5-10mL of CSF in sterile polypropylene tubes for donation to the TIMC-BRAIN biobank. Samples are inverted 3 times and processed on site within 30 minutes of collection.

For peer review only

Sample Processing for TIMC-BRAIN

See Figure 2 for blood and CSF sample processing protocol. Blood and CSF samples are processed on site at TIMC as soon as possible after collection. 0.5mL of whole blood is pipetted from one of the EDTA tubes and stored in a sterile polypropylene cryovial for later DNA analysis. 1mL of blood is removed from the Lithium Heparin tube and stored in 2 x 0.5mL aliqouts with 0.5mL of Cytodelics™ whole blood stabiliser for potential immune cell analysis by flow cytometry (51). Following this, the remaining blood samples (2 x EDTA tubes, 1 x Serum Clot Activator tubes) are centrifuged at 1.8 x g for 10 minutes. Plasma and Serum are subsequently aliquoted in 0.5mL sterile cryovials, labelled with anonymous TIMC-BRAiN participant ID and stored alongside whole blood and Cytodelics™ aliquots at -80°C for future analysis.

< Insert Figure 2 >

CSF samples are centrifuged at 400 x g for 10 minutes at 4°C to pellet immune cells. Cell-free CSF is subsequently aliquoted into 0.5mL sterile cryovials and stored at -80°C for future analysis. The remaining pellet is resuspended in 0.9mL Recovery Cell Culture Freezing Medium (ThermoScientific™) and stored overnight in a Mr Frosty™ Freezing Container at -80°C with aliquots removed and stored separately the following day for future use. This protocol is based on previously published reports examining immune cell composition (52, 53).

Data Storage

For each participant a unique anonymous TIMC-BRAiN ID will be generated which links clinical/neuroimaging data and biological samples. The clinical and cognitive data obtained will be recorded on a dedicated TIMC-BRAiN CRF once a final biobank diagnosis has been applied. A small subset of variables are also recorded for date of blood/CSF sampling and storage in the biobank on a study Sample Storage Form. The CRF is completed by a study investigator/sub-investigator following final biobank diagnosis and aims to capture all data obtained as part of routine clinical assessment as outlined above. Data will be stored using RedCAP (Research Electronic Data Capture), a web

For peer review only

application for building and managing online surveys and databases for research studies. An institutional RedCAP system, protected by host and institutional firewalls, will be maintained at TUH by the TIMC-BRAIN study team. Only the Principal Investigator (PI), Study Investigators/Sub-Investigators and Biobank Manager will have complete access to the TIMC-BRAIN database on RedCAP. Biological samples which are coded matching the SSF on RedCAP are stored in dedicated freezer space in the Meath Foundation Laboratory, TUH. As part of the TIMC-BRAiN consent procedure, participants give permission for storage of data for an initial period of 5 years, which may be extended indefinitely according to review by the TIMC-BRAIN steering committee.

Longitudinal Data

As part of routine care at TIMC participants diagnosed with MCI or dementia are routinely followed up in post-diagnostic support pathways. As part of this, all participants are regularly reviewed with routine repeat cognitive/neuropsychological assessment and dementia severity rating at 18-months. This data will be available for TIMC-BRAiN participants electing to have their clinical data stored in the TIMC-BRAiN repository. A follow-up CRF will document change in cognitive/neuropsychological test scores in addition to progression to dementia/progression of dementia severity.

Withdrawal Procedure

If a participant wishes to withdraw from the study at any stage, this decision is clearly documented by the TIMC-BRAIN investigator/sub-investigator in the patient’s medical notes and CRF. Participants clinical data will be fully removed from the RedCAP database including CRF and SSF. Biological samples donated will be identified by the biobank manager and destroyed. Participants will be informed that the decision to withdraw from TIMC-BRAiN will not affect their ongoing care.

For peer review only

Oversight

The TIMC-BRAiN biobank is guided by a steering committee with a full meeting convened four times per year and reviews any requests for collaboration/sample use in addition to reviewing the day-to-day operating procedures of the biobank.

Data and Sample Access

Applications for access to clinical data/neuroimaging data or biological samples from the TIMC-BRAIN biobank are considered by the Biobank committee and granted for specific reasons. Applications are reviewed once per quarter at the committee meeting and are reviewed, held pending further information/discussion or rejected based on study feasibility or quality, keeping in mind efficient use of the finite sample repository of TIMC-BRAIN and avoiding duplication of research effort. Following request approval, samples and accompanying clinical/neuroimaging data are dispensed to the requesting study team via the Biobank Manager and recorded on the TIMC-BRAIN recruitment log. For transfer of samples and data outside of the host institution (TUH), a Materials Transfer Agreement (MTA) will be generated, reviewed at the Biobank committee meeting and signed by both institutions.

Public and Patient Involvement

Despite the fact that the majority of older adults presenting to geriatric medicine services are interested in participating in research (54), older adults are typically under-represented in cohort studies and clinical trials, with many imposing arbitrary age-related or medical comorbidity-based cut-offs (55). To ensure the relevance, acceptability and feasibility of the TIMC-BRAiN biobank, feedback on the protocol design was obtained from patient representatives undergoing diagnostic work-up in the TIMC MASS. Additionally, a patient representative sits on the TIMC-BRAiN steering committee which discusses the ongoing use of research samples, questions to be addressed and day-to-day running of the biobank.

For peer review only

Ethics and Dissemination

Ethical Approval has been granted by the St James's Hospital (SJH)/Tallaght University Hospital(TUH) Joint Research Ethics Committee (JREC) which operates in compliance with the European Communities (Clinical Trials on Medicinal Products for Human Use) Regulations 2004 and ICH Good Clinical Practice Guidelines (Project ID: 2159). Written informed consent will be obtained from all individuals donating clinical/biological samples to TIMC-BRAiN. A data protection impact assessment and review was performed as part of this application at TUH and TIMC-BRAIN is fully compliant with General Data Protection Regulations (GDPR). It is a key priority for the TIMC-BRAIN Biobank that all research performed using the clinical data and biological sample repository is disseminated in a timely and open access fashion.

Conclusion

Over a five-year period, the TIMC-BRAiN biobank will aim to create a large clinical data and biological sample repository to facilitate basic research aimed at understanding the underlying pathobiology of dementia, the discovery and validation of novel diagnostic and prognostic markers and facilitate research aimed at elucidating potential new treatment options for individuals living with neurodegenerative disease. By creating a central data repository of anonymized clinical data and biological samples, TIMC-BRAiN will enable suitable research questions to be addressed by accessing a ready and comprehensively phenotyped repository. The availability of such a repository will facilitate novel research questions into the underlying aetiology, diagnosis and treatment of neurodegenerative disease and speed-up collaborative research efforts for those living with neurodegenerative disease.

With the advent of new CSF and blood biomarkers in neurodegenerative disease, it is envisaged that TIMC-BRAiN will offer an invaluable resource to research projects aimed at further validating these markers across different populations, and at different stages of the disease process. Crucial to this is the real-world nature of the TIMC-BRAiN cohort, comprised of individuals undergoing diagnostic work-up for early cognitive symptoms or memory complaints. This is crucial to understand the real-world

For peer review only

utility of novel biomarkers for neurodegenerative disease as they emerge. Additionally, by providing basic biomedical researchers with access to anonymized patient blood and CSF samples to readily use, TIMC-BRAiN aims to act as a catalyst in facilitating new insights into the pathobiology of neurodegenerative diseases.

Acknowledgements

The authors wish to acknowledge support from the TIMC-MASS clinical and administrative staff.

Author's Contributions

AHD, HD and SPK are responsible for overall design, administration and conduct of the TIMC-BRAiN biobank. AHD and HD jointly wrote the manuscript. AOC, LM, GS, AM, EKi, CGal, ND, EC, SL, CGaf RE, CM, JJ, GK, Eke, AF, SOD designed clinical protocols for assessment of participants. AHD, HD, COF, NMB, SPK designed the laboratory protocols. All authors have read and approved the final manuscript. All authors were involved in informing the aims and design of the study.

Funding

The TIMC-BRAiN Biobank, AHD, HD and SPK are funded by the Meath Foundation, Tallaght University Hospital. AHD has been awarded the Irish Clinical Academic Training (ICAT) Programme, supported by the Wellcome Trust and the Health Research Board (Grant Number 203930/B/16/Z), the Health Service Executive, National Doctors Training and Planning and the Health and Social Care, Research and Development Division, Northern Ireland.

Competing Interests Statement

None Declared

Patient Consent for Publication

Not Required

For peer review only

Figure Legends

Figure 1. Recruitment Workflow for the Tallaght Institute of Memory and Cognition Biobank for Research in Ageing and Neurodegeneration. Individuals are referred to the TIMC-Memory Assessment and Support Service (MASS) for concerns over cognition or memory symptoms and are first assessed by an Advanced Nurse Practitioner (ANP) in memory. For TIMC-BRAiN, participants undergoing assessment will be provided with a Patient Information Leaflet (PIL) to consider donating clinical and/or biological samples to the TIMC-BRAiN biobank study. Should individuals wish to participate, they will undergo informed consent and may donate biological samples at time of diagnostic lumbar puncture or alongside routine phlebotomy. Participants are followed-up at 18-months to examine for conversion to examine for clinical progression. TIMC-MASS: Tallaght Institute of Memory and Cognition Memory Assessment and Support Service; ANP: Advanced Nurse Practitioner; PIL: Patient Information Leaflet.

Figure 2. Biological Sample Processing for the Tallaght Institute of Memory and Cognition Biobank for Research in Ageing and Neurodegeneration. Biological samples are obtained either alongside routine phlebotomy (for blood samples) or at time of diagnostic lumbar puncture (for cerebrospinal fluid where performed). EDTA: Ethylene-diamine-tetra-acetic acid; SCA: Serum Clot Activator; LiHep: Lithium Heparin; DNA: CSF: Cerebrospinal fluid.

For peer review only

References

1. Livingston G, Huntley J, Sommerlad A, Ames D, Ballard C, Banerjee S, et al. Dementia prevention, intervention, and care: 2020 report of the Lancet Commission. Lancet. 2020;396(10248):413-46.
2. Satizabal CL, Beiser AS, Chouraki V, Chêne G, Dufouil C, Seshadri S. Incidence of Dementia over Three Decades in the Framingham Heart Study. N Engl J Med. 2016;374(6):523-32.
3. Hampel H, Gao P, Cummings J, Toschi N, Thompson PM, Hu Y, et al. The foundation and architecture of precision medicine in neurology and psychiatry. Trends Neurosci. 2023.
4. Hampel H, Shaw LM, Aisen P, Chen C, Lleó A, Iwatsubo T, et al. State-of-the-art of lumbar puncture and its place in the journey of patients with Alzheimer's disease. Alzheimers Dement. 2022;18(1):159-77.
5. Dubois B, Villain N, Frisoni GB, Rabinovici GD, Sabbagh M, Cappa S, et al. Clinical diagnosis of Alzheimer's disease: recommendations of the International Working Group. Lancet Neurol. 2021;20(6):484-96.
6. Briggs R, McHale C, Fitzhenry D, O'Neill D, Kennelly SP. Dementia, disclosing the diagnosis. Qjm. 2018;111(4):215-6.
7. Dubois B, Padovani A, Scheltens P, Rossi A, Dell'Agnello G. Timely Diagnosis for Alzheimer's Disease: A Literature Review on Benefits and Challenges. J Alzheimers Dis. 2016;49(3):617-31.
8. Largent EA, Grill J, O'Brien K, Wolk D, Harkins K, Karlawish J. Testing for Alzheimer Disease Biomarkers and Disclosing Results Across the Disease Continuum. Neurology. 2023.
9. Duits FH, Martinez-Lage P, Paquet C, Engelborghs S, Lleó A, Hausner L, et al. Performance and complications of lumbar puncture in memory clinics: Results of the multicenter lumbar puncture feasibility study. Alzheimers Dement. 2016;12(2):154-63.
10. van Maurik IS, Broulikova HM, Mank A, Bakker ED, de Wilde A, Bouwman FH, et al. A more precise diagnosis by means of amyloid PET contributes to delayed institutionalization, lower mortality, and reduced care costs in a tertiary memory clinic setting. Alzheimers Dement. 2022.
11. de Wilde A, van der Flier WM, Pelkmans W, Bouwman F, Verwer J, Groot C, et al. Association of Amyloid Positron Emission Tomography With Changes in Diagnosis and Patient Treatment in an Unselected Memory Clinic Cohort: The ABIDE Project. JAMA Neurol. 2018;75(9):1062-70.
12. Horie K, Barthélemy NR, Spina S, VandeVrede L, He Y, Paterson RW, et al. CSF tau microtubule-binding region identifies pathological changes in primary tauopathies. Nat Med. 2022;28(12):2547-54.
13. Poggiolini I, Gupta V, Lawton M, Lee S, El-Turabi A, Querejeta-Coma A, et al. Diagnostic value of cerebrospinal fluid alpha-synuclein seed quantification in synucleinopathies. Brain. 2022;145(2):584-95.
14. Hansson O, Edelmayer RM, Boxer AL, Carrillo MC, Mielke MM, Rabinovici GD, et al. The Alzheimer's Association appropriate use recommendations for blood biomarkers in Alzheimer's disease. Alzheimers Dement. 2022;18(12):2669-86.
15. Mattsson N, Palmqvist S, Stomrud E, Vogel J, Hansson O. Staging β-Amyloid Pathology With Amyloid Positron Emission Tomography. JAMA Neurol. 2019;76(11):1319-29.
16. Janelidze S, Mattsson N, Palmqvist S, Smith R, Beach TG, Serrano GE, et al. Plasma P-tau181 in Alzheimer's disease: relationship to other biomarkers, differential diagnosis, neuropathology and longitudinal progression to Alzheimer's dementia. Nat Med. 2020;26(3):379-86.
17. Ashton NJ, Janelidze S, Mattsson-Carlgren N, Binette AP, Strandberg O, Brum WS, et al. Differential roles of Aβ42/40, p-tau231 and p-tau217 for Alzheimer's trial selection and disease monitoring. Nat Med. 2022;28(12):2555-62.
18. O'Connor A, Abel E, Benedet AL, Poole T, Ashton N, Weston PSJ, et al. Plasma GFAP in presymptomatic and symptomatic familial Alzheimer's disease: a longitudinal cohort study. J Neurol Neurosurg Psychiatry. 2023;94(1):90-2.
19. O'Connor A, Karikari TK, Poole T, Ashton NJ, Lantero Rodriguez J, Khatun A, et al. Plasma phospho-tau181 in presymptomatic and symptomatic familial Alzheimer's disease: a longitudinal cohort study. Mol Psychiatry. 2021;26(10):5967-76.
20. Dunne RA, Aarsland D, O'Brien JT, Ballard C, Banerjee S, Fox NC, et al. Mild cognitive impairment: the Manchester consensus. Age Ageing. 2021;50(1):72-80.

For peer review only

21. Ward A, Tardiff S, Dye C, Arrighi HM. Rate of conversion from prodromal Alzheimer's disease to Alzheimer's dementia: a systematic review of the literature. Dement Geriatr Cogn Dis Extra. 2013;3(1):320-32.
22. Hansson O, Seibyl J, Stomrud E, Zetterberg H, Trojanowski JQ, Bittner T, et al. CSF biomarkers of Alzheimer's disease concord with amyloid-β PET and predict clinical progression: A study of fully automated immunoassays in BioFINDER and ADNI cohorts. Alzheimers Dement. 2018;14(11):1470-81.
23. Weiner MW, Veitch DP, Aisen PS, Beckett LA, Cairns NJ, Green RC, et al. The Alzheimer's Disease Neuroimaging Initiative: a review of papers published since its inception. Alzheimers Dement. 2013;9(5):e111-94.
24. Ashton NJ, Janelidze S, Al Khleifat A, Leuzy A, van der Ende EL, Karikari TK, et al. A multicentre validation study of the diagnostic value of plasma neurofilament light. Nat Commun. 2021;12(1):3400.
25. Leuzy A, Janelidze S, Mattsson-Carlgren N, Palmqvist S, Jacobs D, Cicognola C, et al. Comparing the Clinical Utility and Diagnostic Performance of CSF P-Tau181, P-Tau217, and P-Tau231 Assays. Neurology. 2021;97(17):e1681-e94.
26. Leuzy A, Smith R, Cullen NC, Strandberg O, Vogel JW, Binette AP, et al. Biomarker-Based Prediction of Longitudinal Tau Positron Emission Tomography in Alzheimer Disease. JAMA Neurol. 2022;79(2):149-58.
27. Pavlik VN, Burnham SC, Kass JS, Helmer C, Palmqvist S, Vassilaki M, et al. Connecting Cohorts to Diminish Alzheimer's Disease (CONCORD-AD): A Report of an International Research Collaboration Network. J Alzheimers Dis. 2022;85(1):31-45.
28. Bruno D, Schurmann Vignaga S. Addenbrooke's cognitive examination III in the diagnosis of dementia: a critical review. Neuropsychiatr Dis Treat. 2019;15:441-7.
29. Dubois B, Slachevsky A, Litvan I, Pillon B. The FAB: a Frontal Assessment Battery at bedside. Neurology. 2000;55(11):1621-6.
30. Randolph C, Tierney MC, Mohr E, Chase TN. The Repeatable Battery for the Assessment of Neuropsychological Status (RBANS): preliminary clinical validity. J Clin Exp Neuropsychol. 1998;20(3):310-9.
31. Morris JC. The Clinical Dementia Rating (CDR): current version and scoring rules. Neurology. 1993;43(11):2412-4.
32. Galvin JE, Roe CM, Powlishta KK, Coats MA, Muich SJ, Grant E, et al. The AD8: a brief informant interview to detect dementia. Neurology. 2005;65(4):559-64.
33. Wear HJ, Wedderburn CJ, Mioshi E, Williams-Gray CH, Mason SL, Barker RA, et al. The Cambridge Behavioural Inventory revised. Dement Neuropsychol. 2008;2(2):102-7.
34. Zigmond AS, Snaith RP. The hospital anxiety and depression scale. Acta Psychiatr Scand. 1983;67(6):361-70.
35. Buysse DJ, Reynolds CF, 3rd, Monk TH, Berman SR, Kupfer DJ. The Pittsburgh Sleep Quality Index: a new instrument for psychiatric practice and research. Psychiatry Res. 1989;28(2):193-213.
36. Bohannon RW. Reference values for the timed up and go test: a descriptive meta-analysis. J Geriatr Phys Ther. 2006;29(2):64-8.
37. Rockwood K, Song X, MacKnight C, Bergman H, Hogan DB, McDowell I, et al. A global clinical measure of fitness and frailty in elderly people. Cmaj. 2005;173(5):489-95.
38. Vellas B, Guigoz Y, Garry PJ, Nourhashemi F, Bennahum D, Lauque S, et al. The Mini Nutritional Assessment (MNA) and its use in grading the nutritional state of elderly patients. Nutrition. 1999;15(2):116-22.
39. Fazekas F, Barkhof F, Wahlund LO, Pantoni L, Erkinjuntti T, Scheltens P, et al. CT and MRI rating of white matter lesions. Cerebrovasc Dis. 2002;13 Suppl 2:31-6.
40. Duara R, Loewenstein DA, Potter E, Appel J, Greig MT, Urs R, et al. Medial temporal lobe atrophy on MRI scans and the diagnosis of Alzheimer disease. Neurology. 2008;71(24):1986-92.
41. Koedam EL, Lehmann M, van der Flier WM, Scheltens P, Pijnenburg YA, Fox N, et al. Visual assessment of posterior atrophy development of a MRI rating scale. Eur Radiol. 2011;21(12):2618-25.
42. Crutch SJ, Schott JM, Rabinovici GD, Murray M, Snowden JS, van der Flier WM, et al. Consensus classification of posterior cortical atrophy. Alzheimers Dement. 2017;13(8):870-84.
43. Ossenkoppele R, Singleton EH, Groot C, Dijkstra AA, Eikelboom WS, Seeley WW, et al. Research Criteria for the Behavioral Variant of Alzheimer Disease: A Systematic Review and Meta-analysis. JAMA Neurol. 2022;79(1):48-60.

For peer review only - http://bmjopen.bmj.com/site/about/guidelines.xhtml

44. McKeith IG, Boeve BF, Dickson DW, Halliday G, Taylor JP, Weintraub D, et al. Diagnosis and management of dementia with Lewy bodies: Fourth consensus report of the DLB Consortium. Neurology. 2017;89(1):88-100.
45. Rascovsky K, Hodges JR, Knopman D, Mendez MF, Kramer JH, Neuhaus J, et al. Sensitivity of revised diagnostic criteria for the behavioural variant of frontotemporal dementia. Brain. 2011;134(Pt 9):2456-77.
46. Younes K, Borghesani V, Montembeault M, Spina S, Mandelli ML, Welch AE, et al. Right temporal degeneration and socioemotional semantics: semantic behavioural variant frontotemporal dementia. Brain. 2022;145(11):4080-96.
47. Armstrong MJ, Litvan I, Lang AE, Bak TH, Bhatia KP, Borroni B, et al. Criteria for the diagnosis of corticobasal degeneration. Neurology. 2013;80(5):496-503.
48. Street D, Jabbari E, Costantini A, Jones PS, Holland N, Rittman T, et al. Progression of atypical parkinsonian syndromes: PROSPECT-M-UK study implications for clinical trials. Brain. 2023.
49. Höglinger GU, Respondek G, Stamelou M, Kurz C, Josephs KA, Lang AE, et al. Clinical diagnosis of progressive supranuclear palsy: The movement disorder society criteria. Mov Disord. 2017;32(6):853-64.
50. Sachdev P, Kalaria R, O'Brien J, Skoog I, Alladi S, Black SE, et al. Diagnostic criteria for vascular cognitive disorders: a VASCOG statement. Alzheimer Dis Assoc Disord. 2014;28(3):206-18.
51. Nguyen NA, Huang X, Cabrera LE, Pekkarinen PT, Nowlan K, Strandin T, et al. A comprehensive assessment of four whole blood stabilizers for flow-cytometric analysis of leukocyte populations. Cytometry A. 2022.
52. Oh H, Leventhal O, Channappa D, Henderson VW, Wyss-Coray T, Lehallier B, et al. Methods to investigate intrathecal adaptive immunity in neurodegeneration. Mol Neurodegener. 2021;16(1):3.
53. Piehl N, van Olst L, Ramakrishnan A, Teregulova V, Simonton B, Zhang Z, et al. Cerebrospinal fluid immune dysregulation during healthy brain aging and cognitive impairment. Cell. 2022;185(26):5028-39.e13.
54. Davey N, Connolly E, McFeely A, Bambrick P, Pope G, Mulcahy R, et al. 286 PERCEPTIONS OF RESEARCH AMONGST OLDER ADULTS. Age and Ageing. 2022;51(Supplement_3).
55. Goodwin VA, Low MSA, Quinn TJ, Cockcroft EJ, Shepherd V, Evans PH, et al. Including older people in health and social care research: best practice recommendations based on the INCLUDE framework. Age and Ageing. 2023;52(6).

For peer review only

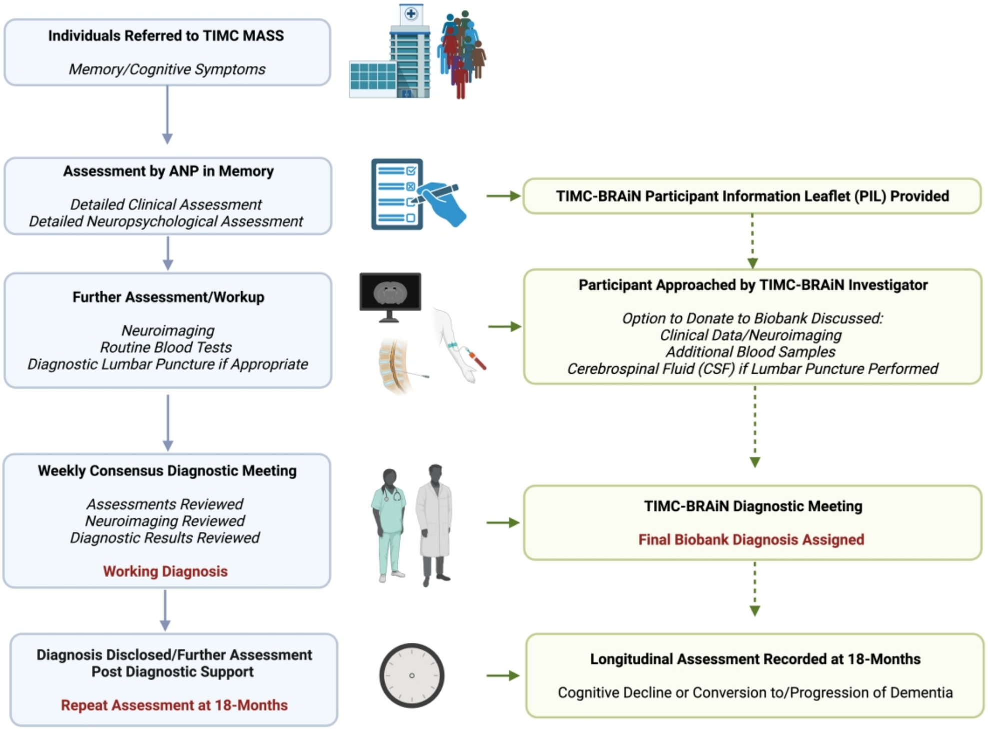

Figure 1. Recruitment Workflow for the Tallaght Institute of Memory and Cognition Biobank for Research in Ageing and Neurodegeneration. Individuals are referred to the TIMC-Memory Assessment and Support Service (MASS) for concerns over cognition or memory symptoms and are first assessed by an Advanced Nurse Practitioner (ANP) in memory. For TIMC-BRAiN, participants undergoing assessment will be provided with a Patient Information Leaflet (PIL) to consider donating clinical and/or biological samples to the TIMC-BRAiN biobank study. Should individuals wish to participate, they will undergo informed consent and may donate biological samples at time of diagnostic lumbar puncture or alongside routine phlebotomy. Participants are followed-up at 18-months to examine for conversion to examine for clinical progression. TIMC-MASS: Tallaght Institute of Memory and Cognition Memory Assessment and Support Service; ANP: Advanced Nurse Practitioner; PIL: Patient Information Leaflet

237x174mm (300 x 300 DPI)

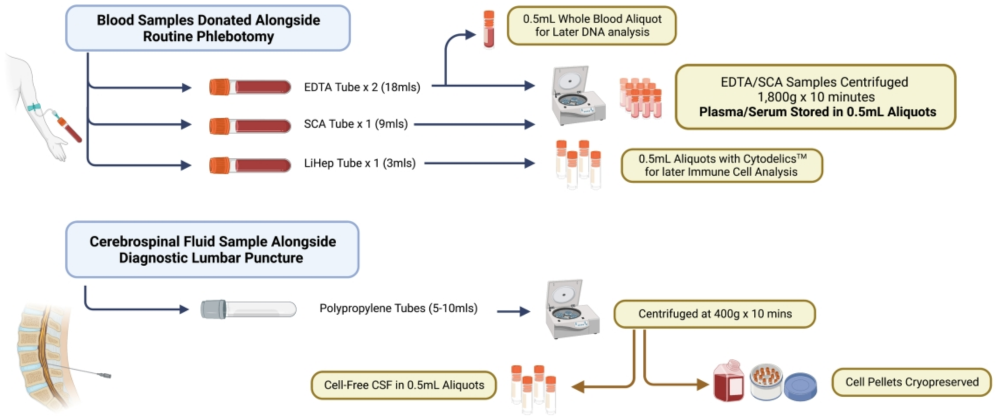

Figure 2. Biological Sample Processing for the Tallaght Institute of Memory and Cognition Biobank for Research in Ageing and Neurodegeneration. Biological samples are obtained either alongside routine phlebotomy (for blood samples) or at time of diagnostic lumbar puncture (for cerebrospinal fluid where performed). EDTA: Ethylene-diamine-tetra-acetic acid; SCA: Serum Clot Activator; LiHep: Lithium Heparin; DNA: CSF: Cerebrospinal fluid.

244x109mm (300 x 300 DPI)

BMJ Open

Protocol for the Tallaght University Hospital Institute for Memory and Cognition – Biobank for Research in Ageing and Neurodegeneration (TIMC-BRAiN)

Journal:	*BMJ Open*
Manuscript ID	bmjopen-2023-077772.R1
Article Type:	Protocol
Date Submitted by the Author:	03-Nov-2023
Complete List of Authors:	Dyer, Adam; Tallaght University Hospital, Institute of Memory and Cognition; Trinity College Dublin, Department of Medical Gerontology, School of Medicine<br>Dolphin, Helena; Tallaght University Hospital, Institute of Memory and Cognition; Trinity College Dublin, Department of Medical Gerontology, School of Medicine<br>O'Connor, Antoinette; Tallaght University Hospital, Department of Neurology<br>Morrison, Laura; Tallaght University Hospital, Institute of Memory and Cognition; Trinity College Dublin, Department of Medical Gerontology, School of Medicine<br>Sedgwick, Gavin; Tallaght University Hospital, Institute of Memory and Cognition<br>McFeely, Aoife; Tallaght University Hospital, Institute of Memory and Cognition; Trinity College Dublin, Department of Medical Gerontology, School of Medicine<br>Killeen, Emily; Tallaght University Hospital, Institute of Memory and Cognition<br>Gallagher, Conal; Tallaght University Hospital, Institute of Memory and Cognition<br>Davey, Naomi; Tallaght University Hospital, Institute of Memory and Cognition<br>Connolly, Eimear; Tallaght University Hospital, Institute of Memory and Cognition<br>Lyons, Shane; Tallaght University Hospital, Department of Neurology<br>Young, Conor; Tallaght University Hospital, Institute of Memory and Cognition<br>Gaffney, Christine; Tallaght University Hospital, Department of Neurology<br>Ennis, Ruth; Tallaght University Hospital, Institute of Memory and Cognition<br>McHale, Cathy; Tallaght University Hospital, Institute of Memory and Cognition<br>Joseph, Jasmine; Tallaght University Hospital, Institute of Memory and Cognition<br>Knight, Graham; Tallaght University Hospital, Institute of Memory and Cognition<br>Kelly, Emmet; Tallaght University Hospital, Institute of Memory and Cognition

	O'Farrelly, Cliona; Trinity College Dublin, Comparative Immunology<br>Bourke, Nollaig M; Trinity College Dublin, Department of Medical Gerontology, School of Medicine<br>Fallon, Aoife; Tallaght University Hospital, Institute of Memory and Cognition; Trinity College Dublin, Department of Medical Gerontology, School of Medicine<br>O'Dowd, Sean; Tallaght University Hospital, Department of Neurology; Trinity College Dublin, Academic Unit of Neurology<br>Kennelly, Sean P; Trinity College Dublin, Department of Medical Gerontology, School of Medicine; Tallaght University Hospital, Institute of Memory and Cognition
**Primary Subject Heading**:	Geriatric medicine
Secondary Subject Heading:	Neurology
Keywords:	Dementia < NEUROLOGY, Delirium & cognitive disorders < PSYCHIATRY, GERIATRIC MEDICINE

SCHOLARONE™
Manuscripts

Protocol for the Tallaght University Hospital Institute for Memory and Cognition – Biobank for Research in Ageing and Neurodegeneration (TIMC-BRAiN)

Adam H Dyer[1-2†], Helena Dolphin[1-2*†], Antoinette O'Connor[3], Laura Morrison[1-2], Gavin Sedwick[1], Aoife McFeely[1-2], Emily Killeen[1], Conal Gallagher[1], Naomi Davey[1], Eimear Connolly[1], Conor Young[1], Shane Lyons[3], Christine Gaffney[3], Ruth Ennis[1], Cathy McHale[1], Jasmine Joseph[1], Graham Knight[1], Emmet Kelly[3], Cliona O'Farrelly[5], Nollaig M Bourke[2], Aoife Fallon[1,2], Sean O'Dowd[3,4], & Sean P Kennelly[1,2]

1. Institute of Memory and Cognition, Tallaght University Hospital, Dublin, Ireland
2. Department of Medical Gerontology, School of Medicine, Trinity College Dublin, Ireland
3. Department of Neurology, Tallaght University Hospital, Dublin, Ireland
4. Academic Unit of Neurology, Trinity College Dublin, Ireland
5. Comparative Immunology, Trinity Biomedical Sciences Institute, Trinity College Dublin, Ireland

†Authors Contributed Equally to This Work

*Corresponding Author

Dr Adam Dyer

Department of Age-Related Healthcare

Tallaght University Hospital

Dublin, Ireland

E-mail: dyera@tcd.ie

Telephone: 00353-1-4142000

Running Title: TIMC-BRAIN Protocol

Key Words: Alzheimer Disease, Dementia, Cerebrospinal Fluid, Blood-Based Biomarkers, Neuroimaging

Abstract Word Count: 299

Main Text Word Count: 4,189

For peer review only

Abstract

Introduction: Alzheimer's Disease (AD) and other dementias affect >50 million individuals globally and are characterised by broad clinical and biological heterogeneity. Cohort and biobank studies have played a critical role in advancing understanding of disease pathophysiology and in identifying novel diagnostic and treatment approaches. However, further discovery and validation cohorts are required to clarify the real-word utility of new biomarkers, facilitate research into the development of novel therapies and to advance our understanding the clinical heterogeneity and pathobiology of neurodegenerative diseases.

Methods and Analysis: The Tallaght University Hospital Institute for Memory and Cognition Biobank for Research in Ageing and Neurodegeneration (TIMC-BRAiN) will recruit 1,000 individuals over 5 years. Participants, who are undergoing diagnostic work-up in the TIMC Memory Assessment and Support Service (TIMC-MASS), will opt to donate clinical data and biological samples into a biobank. All participants will complete a detailed clinical, neuropsychological and dementia severity assessment (including ACE: Addenbrooke's Cognitive Assessment/RBANS: Repeatable Battery for Assessment of Neuropsychological Status/CDR: Clinical Dementia Rating Scale). Participants undergoing venepuncture/lumbar puncture as part of clinical work-up will be offered the opportunity to donate additional blood (serum/plasma/whole blood) and cerebrospinal fluid (CSF) samples for longitudinal storage in the TIMC-BRAIN biobank. Participants are followed at 18-month intervals for repeat clinical and cognitive assessment. Anonymised clinical data and biological samples will be stored securely in a central repository and used to facilitate future studies concerned with advancing with the diagnosis and treatment of neurodegenerative diseases.

Ethics and Dissemination: Ethical Approval has been granted by the St James's Hospital (SJH)/Tallaght University Hospital (TUH) Joint Research Ethics Committee (JREC) [Project ID: 2159] which operates in compliance with the European Communities (Clinical Trials on Medicinal Products for Human Use) Regulations 2004 and ICH Good Clinical Practice Guidelines. Findings using TIMC-BRAiN will be published in a timely and open-access fashion.

For peer review only

Strengths and Limitations of This Study

- TIMC-BRAiN will prospectively recruit 1,000 individuals undergoing assessment for cognitive symptoms obtaining comprehensive clinical data and biological samples that will be stored in a secure biobank offering a bioresource for future studies into the diagnosis, pathogenesis and management of individuals with cognitive or memory disorders
- A key strength of TIMC-BRAiN is the comprehensive neuropsychological data recorded, the extensive data capture at time of routine clinical assessment paired with biological sampling (including blood and CSF) all conducted in a routine memory assessment and support service – offering a repository of real-world patient samples and data for future studies.
- A further strength is the longitudinal follow-up conducted as routine every 18-months for clinical progression, including progression to established cognitive impairment/dementia in those with early cognitive symptoms and disease severity progression in those with established dementia.
- A key limitation of TIMC-BRAiN is that it is a single-site study, however the Memory Assessment and Support Service (MASS) at Tallaght University Hospital (TUH) accepts national referrals and is the largest MASS in the Republic of Ireland.

For peer review only

Introduction

The global prevalence of dementia is expected to sharply increase over the coming decades, affecting >150 million individuals globally by 2050 (1, 2). Recent decades have seen significant advances in our understanding of the neurobiology of dementia, in particular dementia due to Alzheimer's Disease (AD). However, there remains an urgent need to advance understanding of the pathobiology of all dementia syndromes. Such progress will be critical to the discovery of novel diagnostic and prognostic markers, as well as advancing understanding of their real-world utility. In addition, further understanding of clinical and biological phenotypes and underlying pathophysiological processes is an urgent priority for the field to enable better approaches to personalised prevention and treatment, particularly once Disease Modifying Treatments (DMTs) become available.

Neurodegenerative diseases have high degrees of phenotypic, genetic and pathophysiological heterogeneity with traditional diagnostic paradigms centred on late-stage syndromic classification of disease phenotypes (3). Importantly, personalised approaches to clinical-biological classification incorporating comprehensive assessment of clinical phenotype accompanied by the use of appropriate neuro-imaging, biological sampling (such as cerebrospinal fluid (CSF) biomarkers) and further diagnostic tests serves to map observed clinical phenotypes onto known neurobiological substrates (4, 5). An accurate (and timely) diagnosis has important implications for treatment, advance care planning and is valued by those living with neurodegenerative conditions (6, 7).

Many Memory Assessment and Support Services (MASS) use diagnostic biomarkers to assist in the diagnosis and in prognostication of individuals with cognitive impairment. In AD, CSF markers of amyloid and tau pathology are now frequently employed in many MASS (8). Lumbar Puncture [LP] is typically well-tolerated in these settings (9). Additionally, the recent availability of amyloid and tau Positron Emission Tomography (PET) in certain jurisdictions allows non-invasive assessment of AD pathology (10, 11). Progress in the field of biomarker development has not been solely confined to the field of AD. Promising biomarkers that may aid in the diagnosis of other neurodegenerative disorders are also being developed – for instance the identification of isoform-specific tau species in primary

For peer review only

tauopathies such as Cortico-Basal Degeneration (CBD) and Progressive Supranuclear Palsy (PSP) and the detection of alpha-synuclein in CSF by RT-QuiC in prodromal Parkinson's disease and Dementia with Lewy Bodies (DLB) (12, 13).

One of the most exciting advances in the field of neurodegenerative disorders has been the advent of blood-based biomarkers. At present, blood-based biomarkers– exemplified by research in AD - show remarkable promise, even in pre-symptomatic disease stages (14-19). These advances will be accompanied by significant challenges in the implementation and interpretation of these biomarkers in clinical practice. Importantly, validation of these biomarkers in clinical populations, with consideration of issues such as renal clearance, medical comorbidity and population-specific norms, will require the use of large validation cohorts prior to widespread implementation.

A key priority in dementia research is understanding clinical and biological phenotypes across the disease spectrum, which can aid in accurate diagnosis, prognostication and treatment plans for those affected. For instance, Mild Cognitive Impairment (MCI) is characterised by deficits evident on neuropsychological testing which do not interfere with day-to-day function and may stabilise, progress to dementia or even revert to normal cognition over time (20). Around 5-15% of individuals with MCI progress to dementia annually (21). One of the greatest challenges at present is predicting the variable disease trajectory in those affected by MCI and there is an urgent need for new clinical, diagnostic and biological markers that may indicate a greater likelihood of disease progression. Whilst several notable studies have demonstrated the influence of AD biomarkers on disease progression in MCI, further studies are needed to establish the optimal use of prognostic markers in individuals with early cognitive impairment (22)

The development of large-scale clinical, imaging, genetic and biological repositories and real-world clinical cohorts are crucial to the discovery, identification, validation and standardisation of clinical and biomarker-based assessments for AD and other neurodegenerative conditions (15, 23-26). Longitudinal observational cohort studies are integral to advancing understanding of relationship between clinical and fluid biomarkers, cognition and clinical progression across all neurodegenerative diseases, especially as we enter an era of disease modifying therapies (27). The development of

For peer review only

further large cohorts is essential in facilitating the translation of biomarker-based discoveries into clinical practice and further research into the underlying pathobiology and treatment of neurodegenerative conditions.

The Tallaght University Hospital Institute of Memory and Cognition Biobank for Research in Ageing and Neurodegeneration (TIMC-BRAiN) will create a longitudinal biobank of clinical data and biological samples in individuals undergoing assessment for memory and cognitive symptoms at TIMC-MASS. This biobank will facilitate research into clinical and biological biomarkers, in addition to research studies focused on the underlying neurobiology of MCI, dementia and other neurodegenerative conditions.

For peer review only

Aims

TIMC-BRAiN is a longitudinal biobanking study commencing in January 2023 that will recruit 1,000 individuals attending the TIMC-MASS for workup and assessment of cognitive symptoms. TIMC-BRAiN will biobank longitudinal clinical and neuroimaging data alongside biological samples. This biobank will serve as a repository for future research studies that seek to (i) advance understanding of disease pathobiology, (ii) evaluate the use of diagnostic and prognostic tests/assessments and (iii) improve precision medicine approaches across neurodegenerative diseases.

The aims of TIMC-BRAIN are as follows:

- To create a clinical data, neuroimaging and biological sample repository from individuals being assessed for concerns relating to cognition at the TIMC MASS.
- To record final diagnosis and comprehensive clinical phenotyping in those recruited - including demographic information, medical history, cardiovascular risk factors, family history, specific cognitive symptoms (for instance episodic memory/autobiographical memory, language, facial recognition, topographical memory), neuropsychological symptoms, mobility/gait, sleep, nutrition, mood, frailty, hearing and vision.
- To record neuroimaging results coupled to clinical data consisting of Magnetic Resonance Imaging (MRI) or Computed Tomography (CT) Brain results with scoring of MRI scans for vascular burden, parietal atrophy and medial temporal lobe atrophy in addition to results of nuclear imaging and other scans performed where clinically appropriate.
- To biobank biological samples including peripheral blood (whole blood for DNA, serum, plasma and whole blood stored in blood stabiliser) and CSF– including both CSF supernatant and cryopreserved immune cells.
- To longitudinally track changes in cognition (via repeat cognitive assessment) and conversion to/progression of dementia at subsequent 18-month follow-up visits, performed alongside routine clinical care.

For peer review only

As TIMC-BRAiN is a longitudinal clinical data and biological sample repository, there are no pre-specified hypotheses to be tested. TIMC-BRAiN will afford a ready clinical and biological repository for important research questions to be answered in the future and aims to collaborate widely to enable novel basic biological and translational research aimed at improving diagnosis, prognostication and treatments for those affected by neurodegenerative diseases.

For peer review only

Methods

Study Setting & Design

Tallaght University Hospital (TUH) is a tertiary referral hospital in Dublin, Ireland with a catchment area of nearly 500,000 individuals. The TUH Institute of Memory and Cognition (TIMC) is the largest MASS in the Republic of Ireland and receives referrals from both TUH catchment and nationally for individuals experiencing cognitive symptoms. Approximately 400-500 patients are assessed annually in the TIMC-MASS. Once referred to the service, patients are assessed in the first instance by an Advanced Nurse Practitioner (ANP). Comprehensive assessment includes detailed neuropsychological assessment, medical assessment, routine blood tests and appropriate neuroimaging before each case is individually discussed at a weekly Multi-Disciplinary Team (MDT) consensus meeting where working diagnosis is discussed and further investigations if needed, are advised. We anticipate that TIMC-BRAiN will recruit approximately 200 individuals per year to donate clinical data and biological samples, with an estimated 100 of these coming from the roughly 150 individuals who undergo routine diagnostic lumbar puncture annually (paired CSF and blood) and 100 individuals undergoing assessment without lumbar puncture (blood only) from the roughly 250 assessed annually in our unit.

Inclusion and Exclusion Criteria

TIMC-BRAiN will recruit participants undergoing assessment and workup in the TIMC-MASS (See Figure 1) who are willing to provide biological samples for research. TIMC-BRAiN will recruit a diverse cohort including individuals with different levels of cognitive performance - Subjective Memory Concerns (SMCs), Mild Cognitive Impairment (MCI), and dementia - and a representative array of neurodegenerative diseases - to include AD, Lewy Body Disease (LBD), Frontotemporal Dementia (FTD) and other neurodegenerative conditions. In order to reflect a real-world clinical cohort, participants will not be excluded from TIMC-BRAiN based on age or medical comorbidity. However, individuals with severe systemic illness such as malignancy with limited life expectancy, individuals

For peer review only

with current significant alcohol or substance misuse or those with current significant psychiatric comorbidity will be excluded from participation.

<Insert Figure 1>

Participants will be provided with a Participant Information Leaflet (PIL) at time of routine clinical assessment with the ANP in the TIMC and allowed to reflect on whether they wish to donate their clinical/neuroimaging data and biological samples in the TIMC-BRAiN biobank. Participants are informed that participation is entirely voluntary and will not in any way affect their future clinical care. For those undergoing lumbar puncture, participants are approached by a TIMC-BRAiN investigator/sub-investigator at time of procedure to assess whether they wish their clinical data (obtained at time of assessment with the ANP) or biological samples to be included in the biobank. For those not undergoing lumbar puncture, a separate appointment for phlebotomy only is arranged if individuals wish to part-take and have their clinical data and biological samples (blood) bio-banked. Phlebotomy is incorporated alongside routine phlebotomy where possible to minimise venepuncture. Figure 1 outlines assessment and recruitment pathway. Investigators and Sub-Investigators consist of medical staff (Specialist Registrars, Clinical Fellows and Consultants in Geriatric Medicine and Neurology) who are part of the TIMC and are involved in providing clinical care. It is emphasised to individual participants that their willingness to take part (or not) in TIMC BRAiN does not affect their clinical care in any way.

Clinical and neuropsychological Assessment

Clinical and cognitive assessments are performed by an Advance Nurse Practitioner (ANP) as part of routine care at the TIMC – the contents of the TIMC Case Report Form (CRF) are given in Table 1. Information collected includes background/demographic information, medical history, regular medications, hearing and vision, smoking status (yes/no/previous) and family history (detailed in Table 1).

For peer review only

Table 1. Information Recorded on TIMC-BRAiN Case Report Form [CRF]

Item	Data Recorded
**Demographic Information**	
Age	Age at Assessment; Years
Sex	Biological Sex; Male/Female/Non-Binary
Level of Education	Finished Formal Education; Years
Occupation	Occupation; Free Text
Age Retired	Age at Retirement; Years
Marital Status	Non-Married/Married/Divorced/Widowed
Driving Status	Currently Driving/Ceased Driving/Never Drove
**Medical History**	
Prior Stroke/Transient Ischaemic Attack	Yes/No
Recurrent Syncope	Yes/No
Diabetes Mellitus (5)	History of/on DM medication; Yes/No
Hypertension	History of/on antihypertensive medication; Yes/No
Hypercholesterolaemia	History of/on anti-lipidaemic medication; Yes/No
Ischaemic Heart Disease	Yes/No
Alcohol Excess	Yes/No
Epilepsy	History of/on anti-epileptic medication; Yes/No
Concussion/Prior Head Injury	Yes/No
History of Malignancy	Yes/No
Previous Anxiety	History of/on anxiolytic medication; Yes/No
Previous Depression	History of/on antidepressant medication; Yes/No
Hearing Impairment	Yes/No
Vision Impairment	Yes/No
Smoking Status	Yes/No/Previous Smoker
Family History	Memory Difficulties/Dementia/Alzheimer's Disease/ in a 1st degree relative; Yes/No
Regular Medications	Medications List; Coded Using Anatomic Therapeutic Classification (ATC) system
Anosmia	Yes/No
**Cognitive Symptoms at Presentation**	
Symptom Duration	Duration of Symptoms; in Months
Episodic Memory<br>• Lose Track of Days<br>• Forgetting Appointments<br>• Misplacing Objects<br>• Forgetting to Pay Bills<br>• Forgetting How to Use Appliances<br>• Remembering to Take Medications	Each Item Recorded as Yes/No Indicating Recent Change at Time of Assessment
Autobiographical Memory<br>• Forgetting Past Personal Events	Recorded as Yes/No Indicating Recent Change at Time of Assessment
Language<br>• Word-Finding Difficulties<br>• Shrinkage of Vocabulary<br>• Comprehending Speech<br>• Comprehending Written Information<br>• Ability to Engage in Conversations	Each Item Recorded as Yes/No Indicating Recent Change at Time of Assessment
Recognition<br>• Facial Recognition<br>• Getting Lost in Familiar Areas<br>• Changes in Using Public Transport/Driving	Each Item Recorded as Yes/No Indicating Recent Change at Time of Assessment
Informant Concerns<br>• Issues with Cooking<br>• Issues with Orientation<br>• Issues with Driving<br>• Issues with Medication Compliance<br>• Recent Falls	Each Item Recorded as Yes/No Indicating Recent Change at Time of Assessment
**Cognitive Assessment**	
RBANS:<br>• Index I (Immediate Memory)<br>• Index II (Visuospatial/Constructional)<br>• Index III (Language)<br>• Index IV (Attention)<br>• Index V (Delayed Memory)<br>• Global Score	Repeatable Battery for Assessment of Neuropsychological Status (RBANS) Unadjusted (Raw) Score and Centile Score Computed (Based on Age/Education) Normalisation using Duff Norms
ACE-III<br>• Attention<br>• Memory<br>• Fluency<br>• Language	Addenbrooke's Cognitive Assessment (ACE-III) if Unable to Complete RBANS Domain and Total Scores Recorded

For peer review only

• Visuospatial<br>• Overall Score	
CDR<br>• Memory<br>• Orientation<br>• Judgement & Problem Solving<br>• Community Affairs<br>• Home and Hobbies<br>• Personal Care	Clinical Dementia Rating Scale<br>Global Score and Sum of Boxes Recorded
FAB	Frontal Assessment Battery<br>Total Score Recorded
AD8 Administered to Informant	Ascertain-Dementia 8 Questionnaire (Scored from 8)
CBI-R	Cambridge Behavioural Inventory Revised (CBI-R)
**Mood & Anxiety Assessment**	
HADS-Depression<br>HADS-Anxiety	Hospital Anxiety and Depression Scales<br>Total Score Recorded<br>Probable Depression/Anxiety (Score >10) Recorded
**Further Clinical Assessment**	
PSQI	Pittsburgh Sleep Quality Index (0-21)
TUG	Timed-Up-And-Go Test; Time to Complete in seconds recorded
CFS	Clinical Frailty Scale (CFS) to Assess Frailty
MNA	Mini Nutritional Assessment Score (0-7: Malnourished; 8-11: At Risk; 12-14: Normal)
**Neuroimaging**	
MRI Brain<br>• Fazekas Score<br>• MTA Score<br>• Koedam Score	Adjudicated at MDT Consensus Meeting by a panel of >2 geriatricians/neurologists<br>• Fazekas Score 0-3 for White Matter Disease<br>• Medial Temporal Atrophy (MTA) score 0-4<br>• Koedam Score for Parietal Atrophy score 0-3
CT Brain Results	CT Brain results if performed; free text
FDG-PET Results	FDG-PET results if performed; free text
DAT Results	DAT scan results if performed; free text
**Blood Test Results**	
Haematology Results<br>• Full Blood Count (FBC)	Any abnormalities detected; yes/no; recorded in standard units as per hospital laboratory
Biochemistry & Other Results<br>• Renal Profile<br>• Liver Profile<br>• Bone Profile<br>• HbA1c<br>• Lipid Profile<br>• Micronutrients: Vitamin B12, Folate<br>• Vitamin D	Any abnormalities detected; yes/no; recorded in standard units as per hospital laboratory
**Cerebrospinal Fluid Results**	
Diagnostic Lumbar Puncture Results<br>• $A\beta_{1-42}$<br>• T-Tau<br>• P-Tau	All recorded as pg/mL using Roche Elecsys Electrochemiluminesence Assay
**Working (Consensus Meeting) Diagnosis**	
Working Diagnosis at Weekly MDT Meeting	Free Text
**Final (Biobank) Diagnosis**	
Final Diagnosis Adjudicated Following all Investigations	Functional Status:<br>• Subjective Memory Complaints<br>• Mild Cognitive Impairment<br>• Dementia<br><br>Aetiological Diagnosis:<br>• Alzheimer's Disease (amnestic, behavioural, LPA, CBS, and PCA variants)<br>• Lewy Body Disease<br>• Frontotemporal Dementia (FTD) (bvFTD/ nfvPPA/ svPPA)<br>• FTD overlap syndromes: FTD-ALS/FTD-CBS/FTD-PSP<br>• Cortico-basal Syndrome: 4R Tauopathy<br>• Progressive Supranuclear Palsy<br>• Vascular Cognitive Impairment/Dementia<br>• Other Diagnosis [Free Text]<br>• Genetic Diagnosis [Free Text]

LPA: Logopenic variant aphasia, CBS: corticobasal syndrome, PCA: Posterior cortical atrophy; bvFTD: behavioural variant FTD; nfvPPA: non fluent agrammatic PPA, svFTD: semantic variant FTD; 4R Tau: 4 repeat tauopathy: PPA: primary progressive aphasia

For peer review only

All participants undergo comprehensive clinical history, a multi-domain cognitive assessment and a collateral history is obtained for all participants which specifically examines driving safety, medication compliance in addition to classifying duration and domains of cognitive change. Cognitive symptoms are assessed by recent changes in the following domains: (i) episodic memory, (ii) autobiographical memory (iii) language and (iv) facial recognition/topographical memory. All of these variables are recorded as yes/no to indicate a recent change. Total duration of symptoms (in months) and first predominant symptom is also recorded.

Neuropsychological assessment consists of the Addenbrooke's Cognitive Examination (ACE-III) (28) and Frontal Assessment Battery (FAB) (29) – these items are completed in all participants Additionally the Repeatable Battery for the Assessment of Neuropsychological Status (RBANS) is performed if possible (30). The Clinical Dementia Rating Scale (CDR) global/sum of boxes is used to assess for dementia severity (31). The Ascertain Dementia 8 (AD8) questionnaire is also routinely administered to informants (32). The Cambridge Behavioural Inventory (CBI) captures recent behavioural changes and cognitive changes and affective symptoms are reported (33).

Mood and Anxiety are routinely assessed via the Hospital Anxiety and Depression Scale (HADS) Anxiety and Depression scales (34). Sleep is assessed via the Pittsburgh Sleep Quality Index (PSQI) (35). Mobility is assessed by the Timed-Up-And-Go (TUG) test (36). Frailty is assessed via the Clinical Frailty Scale (CFS) (37) . Nutrition is assessed in all participants using the Mini-Nutritional Assessment (MNA) (38). The results of all these assessments are recorded in the TIMC-BRAIN Case Report Form.

Neuroimaging

All individuals assessed in the TIMC have an MRI Brain performed, unless contra-indicated. MRI scans are reviewed at weekly consensus meeting and the following scores applied:

(i) **Fazekas Score**: grades white matter disease, scored from 0-3 (39)

For peer review only

(ii) **Medial Temporal Lobe Atrophy (MTL):** grades mesio-temporal atrophy, scored from 0-4 (40)

(iii) **Parietal Atrophy Score (Koedam Score):** grades parietal atrophy, scored from 0-3 (41)

Scores are applied by a panel of at least two consultants in geriatric medicine and neurology. These scores are recorded in TIMC-BRAiN data repository. Some individuals proceed to having additional imaging e.g. fluorodeoxyglucose (FDG)-PET or Dopamine Uptake (DaTScan) scans. The outcome of all relevant imaging is documented within the data repository (See Table 1).

Diagnostic Workflow in TIMC-MASS & Final TIMC-BRAIN Diagnosis

All cases, after completion of their clinical, neuropsychological and neuroimaging assessments, are discussed at a weekly consensus diagnostic meeting. This meeting, led by a consultant geriatrician/neurologist with expertise in neurodegenerative diseases, determines, where possible, a working (consensus) diagnosis. Further investigations such as advanced neuroimaging or diagnostic CSF sampling are recommended in appropriate cases.

For individuals recruited into TIMC-BRAIN a separate biobank diagnostic meeting occurs on a bi-monthly basis. This meeting is convened by the biobank co-ordinator and attended by the biobank co-ordinator, data manager, research fellows, consultant geriatrician, consultant neurologist and study sub-investigators. Each case is discussed only after all appropriate clinical investigations/assessments have taken place and a final "biobank" diagnosis is confirmed reflecting (i) functional status and (ii) aetiological diagnosis as follows:

Functional status :

- Subjective Memory Complaints (SMCs): individuals with concerns over cognition/memory symptoms, free from established cognitive impairment on neuropsychological testing

For peer review only

- Mild Cognitive Impairment (MCI): impairments on 1 or more neuropsychological domain [typically 1.5 standard deviation fromage/education mean], which do not interfere with day-to-day function. (20)
- Dementia: objective cognitive loss of any domain which is severe enough to cause functional decline in day-to-day activities.

Aetiological Diagnosis:

Published consensus criteria are used to classify aetiology as:

- AD (subtyped into amnestic, behavioural, logopenic variant aphasia, corticobasal syndrome, and posterior cortical atrophy variants) (5, 42, 43)
- Lewy Body Disease (44)
- Frontotemporal Dementia (FTD) subtyped into behavioural variant FTD (bvFTD), semantic variant FTD (svFTD), non-fluent agrammatic FTD (nfvFTD) (45) (46). Also, where applicable, include overlap syndromes: FTD-Corticobasal Syndrome (CBS); FTD-PSP (Progressive Supranuclear Palsy); FTD-ALS (Amytrophic Lateral Sclerosis)
- Corticobasal Syndrome (CBS) – 4R tauopathy (47, 48)
- Progressive Supranuclear Palsy (PSP) (49)
- Vascular Cognitive impairment/Dementia (50)
- Other Diagnosis: This includes individuals with a diagnosis which does not conform to one of the above categories. Details around potential diagnoses will be recorded as free-text by the biobank manager.
- Genetic diagnosis. For the subset of participants with a genetic diagnosis this will also be reordered.

In cases where participants meet criteria for two aetiological diagnosis, e.g. nfvPPA and FTD-PSP, both aetiological diagnoses will be recorded as the final TIMC-BRAIN diagnosis. The biobank co-ordinator is responsible for recording the final biobank diagnosis on the appropriate participants RedCAP diagnostic section (see below under “Data Storage”).

Blood and Cerebrospinal Fluid Sampling

For peer review only

Following informed consent, blood sampling will be performed as part of routine phlebotomy if possible but in certain cases, may need to be performed separately. All blood tests will be obtained between 08:00-12:00 to minimise the risk of diurnal variation on biomarkers and processed on-site at TIMC within 2 hours of blood draw. For TIMC-BRAIN participants, a serum clot activator tube (9mL), two EDTA tubes (9mL each) and a Lithium Heparin (3mL) coated tube are collected. Fasting status will be recorded – however, participants are not asked to fast for the purposes of TIMC-BRAiN participation.

Individuals undergoing diagnostic CSF examination as part of their clinical assessment will be offered the opportunity to donate CSF samples to the TIMC-BRAiN biobank by a study investigator/sub-investigator. LPs are performed between 09:00-12:00 and are carried out in standard aseptic fashion. LPs are performed at the L3-L5 level and manometers are avoided. The maximum number of attempts (including change of operator where required) is 3. Diagnostic samples are obtained in the first instance, followed by an additional 5-10mL of CSF in 2.5mL sterile polypropylene tubes (Starstedt Ltd. Cat no 63.614.625) for donation to the TIMC-BRAIN biobank. CSF is collected by the drip method (gravity drip) as standard. Samples are inverted 3 times and processed on site within 30 minutes of collection.

Sample Processing for TIMC-BRAIN

See Figure 2 for blood and CSF sample processing protocol. Blood and CSF samples are processed on site at TIMC as soon as possible after collection. 0.5mL of whole blood is pipetted from one of the EDTA tubes and stored in a sterile polypropylene cryovial for later DNA analysis. 1mL of blood is removed from the Lithium Heparin tube and stored in 2 x 0.5mL aliqouts with 0.5mL of Cytodelics™ whole blood stabiliser for potential immune cell analysis by flow cytometry (51). Following this, the remaining blood samples (2 x EDTA tubes, 1 x Serum Clot Activator tubes) are centrifuged at 1.8 x g for 10 minutes. Plasma and Serum are subsequently aliquoted in 0.5mL sterile cryovials, labelled with anonymous TIMC-BRAiN participant ID and stored alongside whole blood and Cytodelics™ aliquots at -80°C for future analysis.

For peer review only

< Insert Figure 2 >

CSF samples are centrifuged at 400 x g for 10 minutes at 4ºC to pellet immune cells. Cell-free CSF is subsequently aliquoted into 0.5mL sterile cryovials and stored at -80ºC for future analysis. The remaining pellet is resuspended in 0.9mL Recovery Cell Culture Freezing Medium (ThermoScientific™) and stored overnight in a Mr Frosty™ Freezing Container at -80ºC with aliquots removed and stored separately the following day for future use. This protocol is based on previously published reports examining immune cell composition (52, 53).

Data Storage

For each participant a unique anonymous TIMC-BRAiN ID will be generated which links clinical/neuroimaging data and biological samples. The clinical and cognitive data obtained will be recorded on a dedicated TIMC-BRAiN CRF once a final biobank diagnosis has been applied. A small subset of variables are also recorded for date of blood/CSF sampling and storage in the biobank on a study Sample Storage Form. The CRF is completed by a study investigator/sub-investigator following final biobank diagnosis and aims to capture all data obtained as part of routine clinical assessment as outlined above. Data will be stored using RedCAP (Research Electronic Data Capture), a web application for building and managing online surveys and databases for research studies. An institutional RedCAP system, protected by host and institutional firewalls, will be maintained at TUH by the TIMC-BRAIN study team. Only the Principal Investigator (PI), Study Investigators/Sub-Investigators and Biobank Manager will have complete access to the TIMC-BRAIN database on RedCAP. Biological samples which are coded matching the SSF on RedCAP are stored in dedicated freezer space in the Meath Foundation Laboratory, TUH. As part of the TIMC-BRAiN consent procedure, participants give permission for storage of data for an initial period of 5 years, which may be extended indefinitely according to review by the TIMC-BRAIN steering committee.

For peer review only

Longitudinal Data

As part of routine care at TIMC participants diagnosed with MCI or dementia are routinely followed up in post-diagnostic support pathways. As part of this, all participants are regularly reviewed with routine repeat cognitive/neuropsychological assessment and dementia severity rating at 18-months. This data will be available for TIMC-BRAiN participants electing to have their clinical data stored in the TIMC-BRAiN repository. A follow-up CRF will document change in cognitive/neuropsychological test scores in addition to progression to dementia/progression of dementia severity.

Withdrawal Procedure

If a participant wishes to withdraw from the study at any stage, this decision is clearly documented by the TIMC-BRAIN investigator/sub-investigator in the patient's medical notes and CRF. Participants clinical data will be fully removed from the RedCAP database including CRF and SSF. Biological samples donated will be identified by the biobank manager and destroyed. Participants will be informed that the decision to withdraw from TIMC-BRAiN will not affect their ongoing care.

Oversight

The TIMC-BRAiN biobank is guided by a steering committee with a full meeting convened four times per year and reviews any requests for collaboration/sample use in addition to reviewing the day-to-day operating procedures of the biobank.

Data and Sample Access

Applications for access to clinical data/neuroimaging data or biological samples from the TIMC-BRAIN biobank are considered by the Biobank committee and granted for specific reasons. Applications are reviewed once per quarter at the committee meeting and are reviewed, held pending further information/discussion or rejected based on study feasibility or quality, keeping in mind efficient use of the finite sample repository of TIMC-BRAIN and avoiding duplication of research effort. Following

For peer review only

request approval, samples and accompanying clinical/neuroimaging data are dispensed to the requesting study team via the Biobank Manager and recorded on the TIMC-BRAIN recruitment log. For transfer of samples and data outside of the host institution (TUH), a Materials Transfer Agreement (MTA) will be generated, reviewed at the Biobank committee meeting and signed by both institutions.

Public and Patient Involvement

Despite the fact that the majority of older adults presenting to geriatric medicine services are interested in participating in research (54), older adults are typically under-represented in cohort studies and clinical trials, with many imposing arbitrary age-related or medical comorbidity-based cut-offs (55). To ensure the relevance, acceptability and feasibility of the TIMC-BRAiN biobank, feedback on the protocol design was obtained from patient representatives undergoing diagnostic work-up in the TIMC MASS. Additionally, a patient representative sits on the TIMC-BRAiN steering committee which discusses the ongoing use of research samples, questions to be addressed and day-to-day running of the biobank.

Discussion

Over a five-year period, the TIMC-BRAiN biobank will aim to create a large clinical data and biological sample repository to facilitate basic research aimed at understanding the underlying pathobiology of dementia, the discovery and validation of novel diagnostic and prognostic markers and facilitate research aimed at elucidating potential new treatment options for individuals living with neurodegenerative disease. By creating a central data repository of anonymized clinical data and biological samples, TIMC-BRAiN will enable suitable research questions to be addressed by accessing a ready and comprehensively phenotyped repository. The availability of such a repository will facilitate novel research questions into the underlying aetiology, diagnosis and treatment of neurodegenerative disease and speed-up collaborative research efforts for those living with neurodegenerative disease.

For peer review only

TIMC-BRAiN will recruit 1,000 individuals over five years and obtain comprehensive clinical data and biological samples that will be stored in a secure biobank offering a bioresource for future studies. On of the skey strenghs of TIMC-BRAiN is the comprehensive neuropsychological data recorded, the extensive data capture at time of routine clinical assessment paired with biological sampling (including blood and CSF) all conducted in a routine memory assessment and support service – offering a repository of real-world patient samples and data for future studies. This is crucial in the validation of new biomarkers and diagnostics tests as well as the potential evaluation of new hypothesis around the underlying pathobiology of memory disorders and dementia in older adults. TIMC-BRAiN is also notable for its incorporation alongside clinical care as routine, where follow-up is currently conducted as routine every 18-months for clinical progression, including progression to established cognitive impairment/dementia in those with early cognitive symptoms and disease severity progression in those with established dementia. Whilst TIMC-BRAiN is a single-site study, the Memory Assessment and Support Service (MASS) at Tallaght University Hospital (TUH) accepts national referrals and is the largest MASS in the Republic of Ireland. TIMC-BRAiN is the first biobank embedded within a MASS in the Republic of Ireland.

With the advent of new CSF and blood biomarkers in neurodegenerative disease, it is envisaged that TIMC-BRAiN will offer an invaluable resource to research projects aimed at further validating these markers across different populations, and at different stages of the disease process. Crucial to this is the real-world nature of the TIMC-BRAiN cohort, comprised of individuals undergoing diagnostic work-up for early cognitive symptoms or memory complaints. This is crucial to understand the real-world utility of novel biomarkers for neurodegenerative disease as they emerge. Additionally, by providing basic biomedical researchers with access to anonymized patient blood and CSF samples to readily use, TIMC-BRAiN aims to act as a catalyst in facilitating new insights into the pathobiology of neurodegenerative diseases.

Ethics and Dissemination

Ethical Approval has been granted by the St James's Hospital (SJH)/Tallaght University Hospital(TUH) Joint Research Ethics Committee (JREC) which operates in compliance with the

For peer review only

European Communities (Clinical Trials on Medicinal Products for Human Use) Regulations 2004 and ICH Good Clinical Practice Guidelines (Project ID: 2159). Written informed consent will be obtained from all individuals donating clinical/biological samples to TIMC-BRAiN. A data protection impact assessment and review was performed as part of this application at TUH and TIMC-BRAIN is fully compliant with General Data Protection Regulations (GDPR).

For dissemination of research findings, this will be conducted in several ways. In the first instance, results will be presented at relevant national/international conferences in the areas of geriatric medicine, neurology and memory disorders/dementia. Further, data from studies using data or samples from TIMC-BRAiN will be presented locally and within the host institution. All collaborators who make a meaningful contribution to recruitment, assessment, data curation and management of TIMC-BRAiN will be included on published outputs. Results from studies conducted using the TIMC-BRAiN Biobank will also form part of peer-reviewed journal publications and in doing so it is a key priority for the TIMC-BRAIN Biobank that all papers arising from the biobank are published in an open-access fashion.

Acknowledgements

The authors wish to acknowledge support from the TIMC-MASS clinical and administrative staff.

Author's Contributions

AHD, HD and SPK are responsible for overall design, administration and conduct of the TIMC-BRAiN biobank. AHD and HD jointly wrote the manuscript. AOC, LM, GS, AM, EKi, CGal, ND, EC, SL, CY, CGaf RE, CM, JJ, GK, Eke, AF, SOD designed clinical protocols for assessment of participants. AHD, HD, COF, NMB, SPK designed the laboratory protocols. All authors have read and approved the final manuscript. All authors were involved in informing the aims and design of the study.

Funding

The TIMC-BRAiN Biobank, AHD, HD and SPK are funded by the Meath Foundation, Tallaght University Hospital. AHD has been awarded the Irish Clinical Academic Training (ICAT) Programme, supported by the Wellcome Trust and the Health Research Board (Grant Number 203930/B/16/Z), the

For peer review only

Health Service Executive, National Doctors Training and Planning and the Health and Social Care, Research and Development Division, Northern Ireland.

Competing Interests Statement

None Declared

Patient Consent for Publication

Not Required

Figure Legends

Figure 1. Recruitment Workflow for the Tallaght Institute of Memory and Cognition Biobank for Research in Ageing and Neurodegeneration. Individuals are referred to the TIMC-Memory Assessment and Support Service (MASS) for concerns over cognition or memory symptoms and are first assessed by an Advanced Nurse Practitioner (ANP) in memory. For TIMC-BRAiN, participants undergoing assessment will be provided with a Patient Information Leaflet (PIL) to consider donating clinical and/or biological samples to the TIMC-BRAiN biobank study. Should individuals wish to participate, they will undergo informed consent and may donate biological samples at time of diagnostic lumbar puncture or alongside routine phlebotomy. Participants are followed-up at 18-months to examine for conversion to examine for clinical progression. TIMC-MASS: Tallaght Institute of Memory and Cognition Memory Assessment and Support Service; ANP: Advanced Nurse Practitioner; PIL: Patient Information Leaflet.

Figure 2. Biological Sample Processing for the Tallaght Institute of Memory and Cognition Biobank for Research in Ageing and Neurodegeneration. Biological samples are obtained either alongside routine phlebotomy (for blood samples) or at time of diagnostic lumbar puncture (for cerebrospinal fluid where performed). EDTA: Ethylene-diamine-tetra-acetic acid; SCA: Serum Clot Activator; LiHep: Lithium Heparin; DNA: CSF: Cerebrospinal fluid.

For peer review only

References

1. Livingston G, Huntley J, Sommerlad A, Ames D, Ballard C, Banerjee S, et al. Dementia prevention, intervention, and care: 2020 report of the Lancet Commission. Lancet. 2020;396(10248):413-46.
2. Satizabal CL, Beiser AS, Chouraki V, Chêne G, Dufouil C, Seshadri S. Incidence of Dementia over Three Decades in the Framingham Heart Study. N Engl J Med. 2016;374(6):523-32.
3. Hampel H, Gao P, Cummings J, Toschi N, Thompson PM, Hu Y, et al. The foundation and architecture of precision medicine in neurology and psychiatry. Trends Neurosci. 2023.
4. Hampel H, Shaw LM, Aisen P, Chen C, Lleó A, Iwatsubo T, et al. State-of-the-art of lumbar puncture and its place in the journey of patients with Alzheimer's disease. Alzheimers Dement. 2022;18(1):159-77.
5. Dubois B, Villain N, Frisoni GB, Rabinovici GD, Sabbagh M, Cappa S, et al. Clinical diagnosis of Alzheimer's disease: recommendations of the International Working Group. Lancet Neurol. 2021;20(6):484-96.
6. Briggs R, McHale C, Fitzhenry D, O'Neill D, Kennelly SP. Dementia, disclosing the diagnosis. Qjm. 2018;111(4):215-6.
7. Dubois B, Padovani A, Scheltens P, Rossi A, Dell'Agnello G. Timely Diagnosis for Alzheimer's Disease: A Literature Review on Benefits and Challenges. J Alzheimers Dis. 2016;49(3):617-31.
8. Largent EA, Grill J, O'Brien K, Wolk D, Harkins K, Karlawish J. Testing for Alzheimer Disease Biomarkers and Disclosing Results Across the Disease Continuum. Neurology. 2023.
9. Duits FH, Martinez-Lage P, Paquet C, Engelborghs S, Lleó A, Hausner L, et al. Performance and complications of lumbar puncture in memory clinics: Results of the multicenter lumbar puncture feasibility study. Alzheimers Dement. 2016;12(2):154-63.
10. van Maurik IS, Broulikova HM, Mank A, Bakker ED, de Wilde A, Bouwman FH, et al. A more precise diagnosis by means of amyloid PET contributes to delayed institutionalization, lower mortality, and reduced care costs in a tertiary memory clinic setting. Alzheimers Dement. 2022.
11. de Wilde A, van der Flier WM, Pelkmans W, Bouwman F, Verwer J, Groot C, et al. Association of Amyloid Positron Emission Tomography With Changes in Diagnosis and Patient Treatment in an Unselected Memory Clinic Cohort: The ABIDE Project. JAMA Neurol. 2018;75(9):1062-70.
12. Horie K, Barthélemy NR, Spina S, VandeVrede L, He Y, Paterson RW, et al. CSF tau microtubule-binding region identifies pathological changes in primary tauopathies. Nat Med. 2022;28(12):2547-54.
13. Poggiolini I, Gupta V, Lawton M, Lee S, El-Turabi A, Querejeta-Coma A, et al. Diagnostic value of cerebrospinal fluid alpha-synuclein seed quantification in synucleinopathies. Brain. 2022;145(2):584-95.
14. Hansson O, Edelmayer RM, Boxer AL, Carrillo MC, Mielke MM, Rabinovici GD, et al. The Alzheimer's Association appropriate use recommendations for blood biomarkers in Alzheimer's disease. Alzheimers Dement. 2022;18(12):2669-86.
15. Mattsson N, Palmqvist S, Stomrud E, Vogel J, Hansson O. Staging β-Amyloid Pathology With Amyloid Positron Emission Tomography. JAMA Neurol. 2019;76(11):1319-29.
16. Janelidze S, Mattsson N, Palmqvist S, Smith R, Beach TG, Serrano GE, et al. Plasma P-tau181 in Alzheimer's disease: relationship to other biomarkers, differential diagnosis, neuropathology and longitudinal progression to Alzheimer's dementia. Nat Med. 2020;26(3):379-86.
17. Ashton NJ, Janelidze S, Mattsson-Carlgren N, Binette AP, Strandberg O, Brum WS, et al. Differential roles of Aβ42/40, p-tau231 and p-tau217 for Alzheimer's trial selection and disease monitoring. Nat Med. 2022;28(12):2555-62.
18. O'Connor A, Abel E, Benedet AL, Poole T, Ashton N, Weston PSJ, et al. Plasma GFAP in presymptomatic and symptomatic familial Alzheimer's disease: a longitudinal cohort study. J Neurol Neurosurg Psychiatry. 2023;94(1):90-2.
19. O'Connor A, Karikari TK, Poole T, Ashton NJ, Lantero Rodriguez J, Khatun A, et al. Plasma phospho-tau181 in presymptomatic and symptomatic familial Alzheimer's disease: a longitudinal cohort study. Mol Psychiatry. 2021;26(10):5967-76.
20. Dunne RA, Aarsland D, O'Brien JT, Ballard C, Banerjee S, Fox NC, et al. Mild cognitive impairment: the Manchester consensus. Age Ageing. 2021;50(1):72-80.

For peer review only

21. Ward A, Tardiff S, Dye C, Arrighi HM. Rate of conversion from prodromal Alzheimer's disease to Alzheimer's dementia: a systematic review of the literature. Dement Geriatr Cogn Dis Extra. 2013;3(1):320-32.
22. Hansson O, Seibyl J, Stomrud E, Zetterberg H, Trojanowski JQ, Bittner T, et al. CSF biomarkers of Alzheimer's disease concord with amyloid-β PET and predict clinical progression: A study of fully automated immunoassays in BioFINDER and ADNI cohorts. Alzheimers Dement. 2018;14(11):1470-81.
23. Weiner MW, Veitch DP, Aisen PS, Beckett LA, Cairns NJ, Green RC, et al. The Alzheimer's Disease Neuroimaging Initiative: a review of papers published since its inception. Alzheimers Dement. 2013;9(5):e111-94.
24. Ashton NJ, Janelidze S, Al Khleifat A, Leuzy A, van der Ende EL, Karikari TK, et al. A multicentre validation study of the diagnostic value of plasma neurofilament light. Nat Commun. 2021;12(1):3400.
25. Leuzy A, Janelidze S, Mattsson-Carlgren N, Palmqvist S, Jacobs D, Cicognola C, et al. Comparing the Clinical Utility and Diagnostic Performance of CSF P-Tau181, P-Tau217, and P-Tau231 Assays. Neurology. 2021;97(17):e1681-e94.
26. Leuzy A, Smith R, Cullen NC, Strandberg O, Vogel JW, Binette AP, et al. Biomarker-Based Prediction of Longitudinal Tau Positron Emission Tomography in Alzheimer Disease. JAMA Neurol. 2022;79(2):149-58.
27. Pavlik VN, Burnham SC, Kass JS, Helmer C, Palmqvist S, Vassilaki M, et al. Connecting Cohorts to Diminish Alzheimer's Disease (CONCORD-AD): A Report of an International Research Collaboration Network. J Alzheimers Dis. 2022;85(1):31-45.
28. Bruno D, Schurmann Vignaga S. Addenbrooke's cognitive examination III in the diagnosis of dementia: a critical review. Neuropsychiatr Dis Treat. 2019;15:441-7.
29. Dubois B, Slachevsky A, Litvan I, Pillon B. The FAB: a Frontal Assessment Battery at bedside. Neurology. 2000;55(11):1621-6.
30. Randolph C, Tierney MC, Mohr E, Chase TN. The Repeatable Battery for the Assessment of Neuropsychological Status (RBANS): preliminary clinical validity. J Clin Exp Neuropsychol. 1998;20(3):310-9.
31. Morris JC. The Clinical Dementia Rating (CDR): current version and scoring rules. Neurology. 1993;43(11):2412-4.
32. Galvin JE, Roe CM, Powlishta KK, Coats MA, Muich SJ, Grant E, et al. The AD8: a brief informant interview to detect dementia. Neurology. 2005;65(4):559-64.
33. Wear HJ, Wedderburn CJ, Mioshi E, Williams-Gray CH, Mason SL, Barker RA, et al. The Cambridge Behavioural Inventory revised. Dement Neuropsychol. 2008;2(2):102-7.
34. Zigmond AS, Snaith RP. The hospital anxiety and depression scale. Acta Psychiatr Scand. 1983;67(6):361-70.
35. Buysse DJ, Reynolds CF, 3rd, Monk TH, Berman SR, Kupfer DJ. The Pittsburgh Sleep Quality Index: a new instrument for psychiatric practice and research. Psychiatry Res. 1989;28(2):193-213.
36. Bohannon RW. Reference values for the timed up and go test: a descriptive meta-analysis. J Geriatr Phys Ther. 2006;29(2):64-8.
37. Rockwood K, Song X, MacKnight C, Bergman H, Hogan DB, McDowell I, et al. A global clinical measure of fitness and frailty in elderly people. Cmaj. 2005;173(5):489-95.
38. Vellas B, Guigoz Y, Garry PJ, Nourhashemi F, Bennahum D, Lauque S, et al. The Mini Nutritional Assessment (MNA) and its use in grading the nutritional state of elderly patients. Nutrition. 1999;15(2):116-22.
39. Fazekas F, Barkhof F, Wahlund LO, Pantoni L, Erkinjuntti T, Scheltens P, et al. CT and MRI rating of white matter lesions. Cerebrovasc Dis. 2002;13 Suppl 2:31-6.
40. Duara R, Loewenstein DA, Potter E, Appel J, Greig MT, Urs R, et al. Medial temporal lobe atrophy on MRI scans and the diagnosis of Alzheimer disease. Neurology. 2008;71(24):1986-92.
41. Koedam EL, Lehmann M, van der Flier WM, Scheltens P, Pijnenburg YA, Fox N, et al. Visual assessment of posterior atrophy development of a MRI rating scale. Eur Radiol. 2011;21(12):2618-25.
42. Crutch SJ, Schott JM, Rabinovici GD, Murray M, Snowden JS, van der Flier WM, et al. Consensus classification of posterior cortical atrophy. Alzheimers Dement. 2017;13(8):870-84.
43. Ossenkoppele R, Singleton EH, Groot C, Dijkstra AA, Eikelboom WS, Seeley WW, et al. Research Criteria for the Behavioral Variant of Alzheimer Disease: A Systematic Review and Meta-analysis. JAMA Neurol. 2022;79(1):48-60.

For peer review only

44. McKeith IG, Boeve BF, Dickson DW, Halliday G, Taylor JP, Weintraub D, et al. Diagnosis and management of dementia with Lewy bodies: Fourth consensus report of the DLB Consortium. Neurology. 2017;89(1):88-100.
45. Rascovsky K, Hodges JR, Knopman D, Mendez MF, Kramer JH, Neuhaus J, et al. Sensitivity of revised diagnostic criteria for the behavioural variant of frontotemporal dementia. Brain. 2011;134(Pt 9):2456-77.
46. Younes K, Borghesani V, Montembeault M, Spina S, Mandelli ML, Welch AE, et al. Right temporal degeneration and socioemotional semantics: semantic behavioural variant frontotemporal dementia. Brain. 2022;145(11):4080-96.
47. Armstrong MJ, Litvan I, Lang AE, Bak TH, Bhatia KP, Borroni B, et al. Criteria for the diagnosis of corticobasal degeneration. Neurology. 2013;80(5):496-503.
48. Street D, Jabbari E, Costantini A, Jones PS, Holland N, Rittman T, et al. Progression of atypical parkinsonian syndromes: PROSPECT-M-UK study implications for clinical trials. Brain. 2023.
49. Höglinger GU, Respondek G, Stamelou M, Kurz C, Josephs KA, Lang AE, et al. Clinical diagnosis of progressive supranuclear palsy: The movement disorder society criteria. Mov Disord. 2017;32(6):853-64.
50. Sachdev P, Kalaria R, O'Brien J, Skoog I, Alladi S, Black SE, et al. Diagnostic criteria for vascular cognitive disorders: a VASCOG statement. Alzheimer Dis Assoc Disord. 2014;28(3):206-18.
51. Nguyen NA, Huang X, Cabrera LE, Pekkarinen PT, Nowlan K, Strandin T, et al. A comprehensive assessment of four whole blood stabilizers for flow-cytometric analysis of leukocyte populations. Cytometry A. 2022.
52. Oh H, Leventhal O, Channappa D, Henderson VW, Wyss-Coray T, Lehallier B, et al. Methods to investigate intrathecal adaptive immunity in neurodegeneration. Mol Neurodegener. 2021;16(1):3.
53. Piehl N, van Olst L, Ramakrishnan A, Teregulova V, Simonton B, Zhang Z, et al. Cerebrospinal fluid immune dysregulation during healthy brain aging and cognitive impairment. Cell. 2022;185(26):5028-39.e13.
54. Davey N, Connolly E, McFeely A, Bambrick P, Pope G, Mulcahy R, et al. 286 PERCEPTIONS OF RESEARCH AMONGST OLDER ADULTS. Age and Ageing. 2022;51(Supplement_3).
55. Goodwin VA, Low MSA, Quinn TJ, Cockcroft EJ, Shepherd V, Evans PH, et al. Including older people in health and social care research: best practice recommendations based on the INCLUDE framework. Age and Ageing. 2023;52(6).

For peer review only

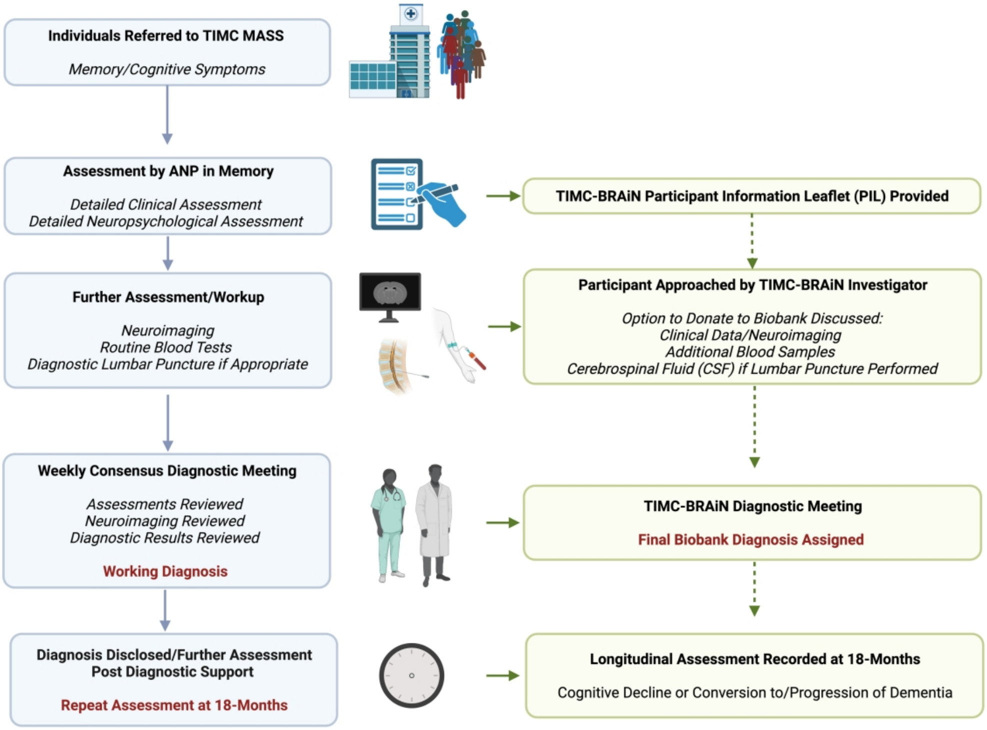

Recruitment Workflow for the Tallaght Institute of Memory and Cognition Biobank for Research in Ageing and Neurodegeneration. Individuals are referred to the TIMC-Memory Assessment and Support Service (MASS) for concerns over cognition or memory symptoms and are first assessed by an Advanced Nurse Practitioner (ANP) in memory. For TIMC-BRAiN, participants undergoing assessment will be provided with a Patient Information Leaflet (PIL) to consider donating clinical and/or biological samples to the TIMC-BRAiN biobank study. Should individuals wish to participate, they will undergo informed consent and may donate biological samples at time of diagnostic lumbar puncture or alongside routine phlebotomy. Participants are followed-up at 18-months to examine for conversion to examine for clinical progression. TIMC-MASS: Tallaght Institute of Memory and Cognition Memory Assessment and Support Service; ANP: Advanced Nurse Practitioner; PIL: Patient Information Leaflet.

237x174mm (300 x 300 DPI)

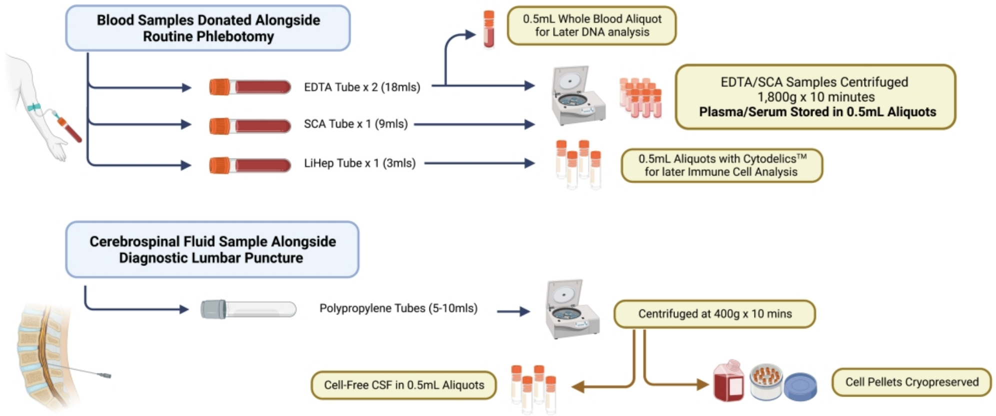

Figure 2. Biological Sample Processing for the Tallaght Institute of Memory and Cognition Biobank for Research in Ageing and Neurodegeneration. Biological samples are obtained either alongside routine phlebotomy (for blood samples) or at time of diagnostic lumbar puncture (for cerebrospinal fluid where performed). EDTA: Ethylene-diamine-tetra-acetic acid; SCA: Serum Clot Activator; LiHep: Lithium Heparin; DNA: CSF: Cerebrospinal fluid.

244x109mm (300 x 300 DPI)

TIMC-BRAiN

TUH Institute for Memory & Cognition – Biobank for Research in Ageing & Neurodegeneration

Participant Information Leaflet (PIL)

Name of Biobank	TIMC-BRAiN Biobank
Location	Tallaght Institute of Memory and Cognition (TIMC), TUH
Principal Investigator (PI)	Professor Sean Kennelly, TUH
Co-Investigators	Dr Adam Dyer, TUH; Dr Helena Dolphin, TUH
Data Controller	Department of Age-Related Healthcare, TUH
Contact	Sean.Kennelly@tuh.ie; Adam.Dyer@tuh.ie; Helena.Dolphin@tuh.ie
Data Protection	Sean.Kennelly@tuh.ie; Adam.Dyer@tuh.ie; Helena.Dolphin@tuh.ie

You are invited to take part in the **TIMC-BRAiN Biobank**. This research study collects **clinical data** (including memory assessments, medical conditions and medications) and **biological samples** (including blood and cerebrospinal fluid samples), donated by participants for health research. This study helps doctors and researchers to learn more about the diagnosis and treatment of memory problems (such as cognitive impairment and dementia). This helps us to better understand how and why people develop these conditions. **Please read this leaflet carefully. Your physician in the Institute of Memory and Cognition in TUH can answer any questions that you may have.**

Key Words

When we say…	We mean…
Academic research	Research carried out in hospitals, universities, colleges and research institutes
Biobank	A collection of clinical data and biological samples donated by participants for health research
DNA	Your DNA (genes) make who you are by instructing cells to carry out specific tasks
Genetic Research	Research which examines peoples' genetic information (genes) to help understand how cognitive impairment and dementia develops and how best to treat it
Clinical Data	Information in your hospital chart, electronic patient record and other hospital databases including name, address, test results, images and scans.
Identifiable data	Information that might identify you such as name, address, date of birth
Non-identifiable ("Coded") data	Information that might identify you has been removed and replaced by a unique code. This is used instead of name, address, date of birth
Principal Investigator	The person responsible for a biobank or specific research study
Research Ethics Committee	Independent group of people who review each study to ensure that research is carried out ethically and safely and that rights are protected
Research studies	Research to learn more about cognitive impairment and dementia such as genetic research, how the condition changes or progresses, early detection and new diagnostics and treatments

For peer review only

PART 1 – THE STUDY

Why is this study being done?

Our research aims to learn more about **why people develop memory problems (such as cognitive impairment and dementia)**. We aim to understand **how and why** certain people develop these conditions. This includes analysis of **clinical information**, blood samples as well as lifestyle factors and family history. Additionally, we are trying to **develop new tests and methods of diagnosing these conditions**. This type of research requires many people to donate samples and data. This study (TIMC-BRAiN) speeds up research by allowing the research team have samples and data ready to use when needed. **The aim of this is to improve the diagnosis, management and treatment** of **memory problems (cognitive impairment** and **dementia).**

Your clinical data and biological samples will be stored **until researchers need them** in order to answer important questions. Your data and samples may thus be included in many different research studies and for this reason your data and samples may be stored indefinitely (forever). This biobank may share your **non-identifiable data** with other researchers working around the world. However, no potentially identifiable data will be shared outside of Tallaght University Hospital.

What happens if I take part ?

If you would like to take part, please read this leaflet and sign the consent form at the end. You will be given a copy of this leaflet to keep. A member of the research team will discuss this with you in more detail. If you are happy to take part, the biobank will collect and store

- **Clinical information** from your medical records, including medication history and medication usage in addition to test results from your cognitive assessment (memory tests)
- **Blood samples:** if you are having a routine blood test, the research doctor may take extra samples of blood to give to the biobank This is a small sample – about 3 tablespoons of blood
- **Cerebrospinal fluid samples**: if you are having a lumbar puncture as part of your memory assessment and diagnosis, the research team may ask to take an extra sample to store in the biobank. **This will only take place if you are having a lumbar puncture performed as part of your clinical assessment. There are no lumbar punctures being carried out solely for research purposes.**
- **Genetic (DNA) Samples**: DNA samples will be extracted from blood and samples for research. DNA is the molecule that provides instructions for how cells in our body work. By looking at DNA from people with and without vasculitis we may see differences that are important for diagnosis, treatment or understanding the condition.

For peer review only

What will happen to my clinical data and biological samples ?

All samples and data will be given a random study number in TUH by your study physician in a process called pseudo-anonymisation or “coding”. This is intended to mask your identity. All biological samples will be stored in the Meath Foundation laboratory in Tallaght University Hospital using this code. Additionally, your clinical data will be stored on secure databases using this code. Only the study investigators have access to this code and this code will not be shared outside of the study team.

Samples will be stored in a freezer and will remain frozen for decades and potentially indefinitely (forever) so that we can identify future diagnostics and treatment for cognitive impairment and dementia. Samples are being kept indefinitely as scientific research rapidly changes and advances all the time, and we do not yet know what kinds of research questions will arise in the future. The only individuals with direct access to your samples are the investigators of the TIMC-BRAiN study.

Data and samples may be shared with other collaborators. If this is the case, a “Materials Transfer Agreement” will be drawn up by the TIMC-BRAiN study co-ordinators and the collaborating institutions/research groups. This may include samples and data being transferred outside of the European Economic Area (EEA). These samples and data will have all identifying features removed.

What are the benefits to me ?

Research can lead to better diagnostic tests, treatments and quality of life for people with cognitive impairment and dementia. The treatments that we have now developed are as a result of past research studies and biobanks. The TIMC biobank collects samples and healthcare data for future research and therefore we cannot predict at this time what research questions will arise in the future and what discoveries these will lead to. It takes many years to do research, so this research may not directly benefit you personally. **However, by participating you are contributing to improving the diagnosis, treatment and management of individuals living with cognitive impairment and dementia long into the future.**

What are the risks of participation ? Will I be informed of my results ?

As with any medical records, there is a theoretical risk of accidental disclosure of healthcare data. However, this is very unlikely as every effort will be made to protect your privacy (see below Section B and Section C). A blood sample may cause bruising or slight discomfort. The additional sample taken at time of diagnostic lumbar puncture poses minimal extra risk (you may be very slightly more likely to experience a post lumbar-puncture headache, however this additional risk is very minimal).

There are additional risks involved in genetic testing. Our genetic testing is entirely focused on the study of the genetics of cognitive impairment and dementia. Therefore it is possible

For peer review only

that even if you take part in the study. Even if we do not discover a genetic cause for your condition now, one may still be discovered in the future. It is common that we discover no genetic association for cognitive impairment or dementia or that it takes many years in order to do so. No results will be communicated as a result of genetic testing in this study as this is entirely a research study aimed at discovering genes for cognitive impairment and dementia. Thus, we are not testing for any other genetic mutations of potential clinical significance.

Do I have to take part ?

No, you do not have to take part. Your decision is completely voluntary and will not affect your medical care now or in the future.

What happens if I change my mind ?

You can change your mind at any time. If you contact the study team at any time to inform us that you no longer wish to participate, your samples will be **removed from the biobank and destroyed**. Additionally, the clinical data contributed as part of the biobanked will be removed from the biobank database. From that point on, none of your further samples of information will be used as part of the biobank. However, it may not be possible to destroy samples and healthcare data that have already been used for research as this could influence the results of the research.

What happens the results from research ?

Research is usually published in scientific and medical journals or presented at conferences so that other doctors and researchers can learn. You will not be identified in any of these publications or presentations. The research from TIMC-BRAiN will go on to inform the diagnosis, investigation and treatment of cognitive impairment and dementia into the future.

PART 2 – BIOBANK MANAGEMENT

How does the TIMC-BRAiN Biobank protect my privacy ?

TIMC-BRAiN has very strict governance procedures in place**. Only the TIMC-BRAiN team** have access to clinical data and biological samples. Only research studies that have been approved by the SJH/TUH JREC Ethics Committee will use clinical data and biological samples. Prof Kennelly and the study team must sign **legal agreements before clinical data and biological samples can be shared.** This ensures that your samples are only used as agreed. All members of the research team will undergo **General Data Protection Regulation (GDPR)** training and are bound by hospital confidentiality rules. TIMC-BRAiN ensures that your data is stored in a de-identified "coded" manner on **secure servers and only accessible by members of the study team**.

For peer review only - http://bmjopen.bmj.com/site/about/guidelines.xhtml

Has the biobank been approved by an ethics committee ?

Yes. This biobank has been approved by the SJH/TUH Joint Research Ethics Committee.

How is this biobank funded ?

This biobank is funded by departmental funds from the **Tallaght Institute of Memory and Cognition, Department of Age-Related Healthcare, Tallaght University Hospital**. The biobank does not make a profit from collecting or sharing your samples and healthcare data for health research.

Will I be paid for my involvement ?

No. You will not be paid to take part. It is hoped that your donation of samples and data will help other individuals undergoing investigation and treatment for cognitive impairment and dementia in the future.

PART 3 – WHAT DOES THE BIOBANK DO WITH MY HEALTHCARE DATA ?

Why does TIMC-BRAiN need to collect my clinical data ?

Clinical data is needed in order for researchers to choose the right biological samples for research or help interpret the results of their studies. To take part, you will be asked to share your clinical data collected as part of memory assessment and clinical workup with the TIMC-BRAiN biobank.

What type of clinical data will be collected ?

The biobank will collect healthcare and clinical data from your hospital chart, electronic records and other hospital databases. This data will only be accessed by study investigators (listed above). The study investigators (Dr Dyer/Dolphin, Prof Kennelly) may know your identity to follow your care and treatment. However, your identifiable information will only be able to be accessed by study investigators. The TIMC-BRAiN biobank will store the following clinical data:

- Name, gender, date of birth, hospital number, laboratory identifiers; this information will be stored on a protected database **<u>only accessible by the study investigators</u>**
- Results of blood tests, medical history, regular medications, lifestyle information (smoking, alcohol use), neuroimaging results and final diagnosis and treatment. This information is all routinely obtained as part of work-up, assessment, diagnosis and treatment of cognitive impairment and dementia.
- Results of cerebrospinal fluid investigations, if applicable.

For peer review only

How is my data stored and shared ?

When you are enrolled in the study, you will be assigned an anonymous study number. The "key" will be stored in TUH on a locked database only accessible to the study investigators. Clinical data and clinical results will be stored on a secure database maintained on servers in Tallaght University Hospital. This will be stored using the REDCap system (Research Electronic Data Capture) which is used globally for the storage of sensitive research data. This will only be accessible by study investigators.

Your clinical data may be shared as:

(i) **Identifiable Data**: the study investigators at the Tallaght Institute of Memory and Cognition may know your identity in order to follow your care and treatment. Only the above named investigators will have access to your identifiable data

(ii) **Non-identifiable data:** researchers working as part of collaborations or research studies outside of the study team (either in Trinity College Dublin, academic partners of TUH, or as part of other researchers) receive "non-identifiable data". This means that information that may identify you has been removed and replaced with a unique code which is used instead of your hospital number, date of birth, address. These researchers work in universities or hospital. Data sharing will only take place as outlined above and your data will not be shared with any external companies or for-profit organisations.

What are my rights ?

TIMC-BRAiN has data protection measures in place and is committed to ensuring that your rights under GDPR (General Data Protection Regulation) are protected. The Department of Age-Related Healthcare is the data controller for this study. We also ask for your consent as a data protection safeguard, in accordance with the Irish Health Research Research Regulations 2018.

- Under the European General Data Protection Regulation (GDPR), the lawful basis for processing your data in this study is for scientific research (Article 9(2) (j)) in the public interest (Article 6(1)(e)).
- Under GDPR, you can exercise the following rights in relation to your personal data, unless the request would make it impossible or very difficult to conduct the research:
    - The right to access to your data and receive a copy of it
    - The right to restrict or object to processing of your data
    - The right to object to any further processing of the information we hold about you (except where it is de-identified)
    - The right to have inaccurate information about you corrected or deleted

For peer review only

- The right to receive your data in a portable format and to have it transferred to another data controller
- The right to request deletion of your data

- You can exercise these rights be contacting your study doctor/study investigator
- Under GDPR, if you are not satisfied with how your data is being used, you have the right to lodge a complaint with the Data Protection Commissioner of Ireland or the study Data Protection Officer (dpo@tuh.ie)

For peer review only

TIMC-BRAiN

TUH Institute for Memory & Cognition – Biobank for Research in Ageing & Neurodegeneration

CONSENT FORM

To be completed by the participant:

I have read and understood the information leaflet for the study. The information has been fully explained to me and I have been able to ask questions, all of which have been answered to my satisfaction	YES ☐	NO ☐
I have had the opportunity to discuss the study, ask questions about the study and I have received satisfactory answers to all my questions.	YES ☐	NO ☐
I understand that my participation is voluntary, and I can withdraw my biological material and data at any time without giving a reason. I understand that opting out will not affect my future medical care.	YES ☐	NO ☐
I understand that sections of my medical notes may be looked at by study investigators at Tallaght Hospital. I give permission for these individuals to access my records. All information will be kept private and confidential.	YES ☐	NO ☐
I agree for the entry of my clinical data into the registry. I give explicit informed consent for my data to be processed as part of this research study. I understand that my data will be securely coded and stored indefinitely.	YES ☐	NO ☐
I agree to provide blood and (if applicable) cerebrospinal fluid samples for this study as described in the information leaflet. I understand that my samples will be securely coded and stored indefinitely. The risk of taking samples has been explained.	YES ☐	NO ☐
I consent for my biological coded samples (including DNA) to be shared with authorised third parties including national and international institutions and academic institutions. I understand that this requires a legal materials transfer agreement between researchers at TUH and these institutions.	YES ☐	NO ☐
I agree to be contacted by researchers as part of this study	YES ☐	NO ☐
I consent to undergo venepuncture for the purposes of this study	YES ☐	NO ☐
I consent to participation in this research study having been fully informed of the risks, benefits and purpose of the study	YES ☐	NO ☐
I consent to be contacted with regard to the possible use of my data and/or biological material for future research studies (with consent)	YES ☐	NO ☐
I understand that processing of my personal data, including transfer of data outside the EU, will be protected in accordance with the General Data Protection Regulation	YES ☐	NO ☐
I understand that results from the analysis of my samples will not be given to me. I understand that there are no direct benefits to me and I will not benefit financially in any way.	YES ☐	NO ☐

Participant's Name (Block Capitals):	
Participant's Signature:	
Date:	

For peer review only

To be completed by the **RESEARCHER**:

I have fully explained the purpose and nature (including benefits and risks) of this study to the participant in a way that he/she could understand. I have invited him/her to ask questions on any aspect of the study.	YES □	NO □
I confirm that I have given a copy of the information leaflet and consent form to the participant and family member/carer.	YES □	NO □

Researcher's Name (Block Capitals):	
Researcher's Title & Qualifications:	
Researcher's Signature:	
Date:	

For peer review only
